# TDP‐43/ALKBH5‐mediated m^6^A modification of CDC25A mRNA promotes glioblastoma growth by facilitating G1/S cell cycle transition

**DOI:** 10.1002/mco2.70108

**Published:** 2025-02-18

**Authors:** Yunxiao Zhang, Sidi Xie, Weizhao Li, Junwei Gu, Xi‐an Zhang, Bowen Ni, Ziyu Wang, Runwei Yang, Haimin Song, Yaxuan Zhong, Peiting Huang, Jinyao Zhou, Yongfu Cao, Jing Guo, Yawei Liu, Songtao Qi, Hai Wang

**Affiliations:** ^1^ Department of Neurosurgery, Nanfang Hospital Southern Medical University Guangzhou China; ^2^ Department of Neurosurgery, Institute of Brain Diseases Nanfang Hospital of Southern Medical University Guangzhou China; ^3^ The First People's Hospital of Xiushui County Jiujiang China; ^4^ Department of Neurosurgery & Medical Research Center, Shunde Hospital Southern Medical University Shunde China; ^5^ Department of Neurosurgery The First Affiliated Hospital of Gannan Medical University Ganzhou China; ^6^ School of the First Clinical Medicine Gannan Medical University Ganzhou China; ^7^ Department of Neurology, Guangdong Provincial People's Hospital Southern Medical University Guangzhou Guangdong China; ^8^ Department of Neurosurgery Dongguan Tungwah Hospital Dongguan China; ^9^ Department of Neurosurgery, Key Laboratory of Biological Targeting Diagnosis, Therapy and Rehabilitation of Guangdong Higher Education Institutes The Fifth Affiliated Hospital of Guangzhou Medical University Guangzhou China; ^10^ Epilepsy Center Guangdong Sanjiu Brain Hospital Guangzhou China

**Keywords:** ALKBH5, CDC25A, glioblastoma, intratumor heterogeneity, TDP‐43

## Abstract

Glioblastoma (GBM) exhibits significant intratumor heterogeneity (ITH), indicating the presence of tumor cells with diverse growth rates. Here, we aimed to identify fast‐growing cells in GBM and elucidate the underlying mechanisms. Precisely targeting these cells could offer an improved treatment option. Our results found that targeting ALKBH5 expression impaired GBM proliferation and tumor stemness. Nuclear but not overall expression of ALKBH5 differs between monoclonal cells derived from the same patient with different proliferation rates. Mechanistically, ALKBH5 interacted with TAR DNA‐binding protein 43 (TDP‐43) in fast‐growing cells. Furthermore, TDP‐43 facilitated the nuclear localization of ALKBH5 and its binding to cell division cycle 25A (CDC25A) pre‐mRNA. The TDP‐43/ALKBH5 complex regulates CDC25A mRNA splicing via N6‐methyladenosine (m^6^A) demethylation to maintain the expression of its oncogenic isoform (CDC25A‐1), ultimately promoting the G1/S phase transition and growth of GBM cells. TRAD01 selectively targeted the interaction between TDP‐43 and ALKBH5, leading to significant antitumor effects both in vitro and in vivo. Our study identified a novel epigenetic mechanism by which TDP‐43/ALKBH5 contributes to GBM growth via m^6^A modification and alternative splicing. Therefore, targeting the TDP‐43/ALKBH5 axis might be a promising therapeutic strategy for GBM patients.

## Graphical abstract

In this study, TDP‐43 interacts with ALKBH5 and promotes its binding to CDC25A pre‐mRNA in fast‐growing GBM cells. TDP‐43/ALKBH5 complex demethylates CDC25A pre‐mRNA, inhibiting its exon 6 skipping and preserving the expression of the oncogenic isoform CDC25A‐1, thereby promoting G1/S phase transition and growth of GBM cells. TRAD01 targeted the interaction between TDP‐43 and ALKBH5, leading to significant antitumor effects. Therefore, targeting the TDP‐43/ALKBH5 axis might be a promising therapeutic strategy for GBM patients.

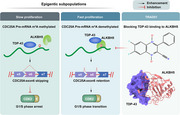

## 1 INTRODUCTION

Glioblastoma (GBM) is the most malignant and prevalent primary brain tumor^1^ and remains almost invariably recurrent and lethal due to inherent heterogeneity,^2^, ^3^ even with maximal surgical resection followed by highly aggressive temozolomide (chemotherapy and radiation treatment.^4^ Consequently, increasing research efforts are focused on the development of novel therapies for GBM.^5^, ^6^ GBM is known for its diverse histological and molecular pathology traits and represents one of the most heterogeneous tumors.^7^, ^8^ Intratumor heterogeneity (ITH) poses significant preclinical and clinical challenges for GBM.^9^, ^10^, ^11^ ITH is a hallmark of cancer that generates phenotypic diversity, promoting malignant progression by allowing the fittest cells to outgrow their counterparts and gain dominance in the tumor microenvironment.^12^ Therefore, targeting the fittest cells is essential for GBM management. Although ITH is usually associated with mutational heterogeneity, recent findings suggest its involvement in epigenetic heterogeneity.^13^ However, the epigenetic mechanisms governing ITH have yet to be fully elucidated.

N6‐methyladenosine (m^6^A) is the most prevalent internal modification on eukaryotic mRNA^14^, ^15^ and participates in RNA splicing, export, translation, and decay.^16^, ^17^ m^6^A is catalyzed by a multicomponent protein complex consisting of the “writers” METTL3 and METTL14 and their cofactor WTAP.^18^, ^19^, ^20^ On the other hand, two demethylases, FTO and ALKBH5, act as “erasers” that reverse the m^6^A modification on mRNA.^21^, ^22^ In addition, YT521‐B homology (YTH) domain‐containing proteins, including YTHDF1‐3, YTHDC1, and YTHDC2, have been recognized as “readers” that specifically identify m^6^A sites.^23^ There is growing evidence that ALKBH5 is an essential regulator of tumor progression and is involved in the malignant progression of several tumor types,^24^ including breast cancer,^25^ GBM,^26^ and acute myeloid leukemia,^27^ by demethylating m^6^A‐modified mRNA.

The utilization of small molecule inhibition technology in the advancement of tumor‐targeted medications shows significant promise.^28^ Glivec stands out as the pioneering small molecule anticancer‐targeted drug available on the market, focusing on tyrosine kinases mutated in specific genes of cancer cells.^29^ Idasanutlin, a potent and selective inhibitor of MDM2, effectively inhibits the protein–protein interactions between MDM2‐p53 and is currently being evaluated in 17 different clinical trials for the treatment of acute myeloid leukemia, neuroblastoma, and estrogen receptor‐positive breast cancer.^30^ The dysregulation of m^6^A modification has been associated with the onset of various diseases.^31^ By interfering with m^6^A demethylases, it is possible to impede tumor growth by regulating m^6^A modification and subsequently influencing the expression of crucial genes.^21^, ^26^ Consequently, the investigation of small molecule inhibitors that target m^6^A demethylases has emerged as a focal point in the realm of antitumor drug research. The introduction of the first small molecule inhibitor of FTO, rhein,^32^ followed by the finding of a selective inhibitor of FTO, meclofenamic acid,^33^ and a more potent small molecule inhibitor, FB‐23,^34^ has laid the groundwork and offered a novel avenue for the targeted treatment of associated diseases. Nonetheless, research on small molecule inhibitors that target the m^6^A demethylase ALKBH5 remains scarce.

In previous studies, ALKBH5 has been implicated in promoting malignant phenotypes such as tumor proliferation, invasion, and migration. However, its regulation of ITH‐mediated tumor phenotypic diversity remains unclear. Our study revealed that the nuclear expression of ALKBH5 rather than its overall expression influences the phenotypic diversity of ITH‐mediated proliferative phenotypic diversity. In this study, we identified the interacting proteins and m^6^A targets of ALKBH5 in fast‐growing GBM cells. Our study presented significant evidence that the TAR DNA‐binding protein 43 (TDP‐43)/ALKBH5 complex contributes to proliferation of fast‐growing GBM cells through its roles in m^6^A modification and alternative splicing. Our study also identified a promising small molecule that prevents the binding of TDP‐43 and ALKBH5, potentially offering a novel treatment option for GBM patients.

## 2 RESULTS

### 2.1 Targeting ALKBH5 expression impairs GBM proliferation and stemness

To investigate the correlation between ALKBH5 expression and GBM prognosis, we conducted a Kaplan–Meier survival analysis of two GBM datasets—one from the Chinese Glioma Genome Atlas (CGGA) and another from Nanfang Hospital (based on immunohistochemistry [IHC] staining score). The results of the analysis revealed that GBM patients with elevated ALKBH5 expression have a worse prognosis (Figure S1A–C and Table S1). We further sought to determine whether ALKBH5 promotes the malignant phenotype of GBM using EdU assays in primary GBM cells (NFHDCD) and U87MG cells. We found that inhibiting ALKBH5 expression using three different siRNAs led to a reduction in cell proliferation (Figure S1D–G). Additionally, transfection with shRNA lentivirus to knock down ALKBH5 in NFHDCD cells resulted in G1/S phase arrest (Figure S1H,I). Conversely, overexpression of ALKBH5 promoted G1/S phase transition in GBM cells (Figure S1J,K). Furthermore, ALKBH5 downregulation significantly decreased expression of SOX2 and the tumor sphere formation frequency of GBM stem‐like cells (GSCs) derived from NFHDCD cells (NFHDCD‐GSCs) (Figure S1L,M).

### 2.2 Nuclear expression of ALKBH5 impacts ITH‐mediated phenotypic diversity in GBM

**FIGURE 1:**
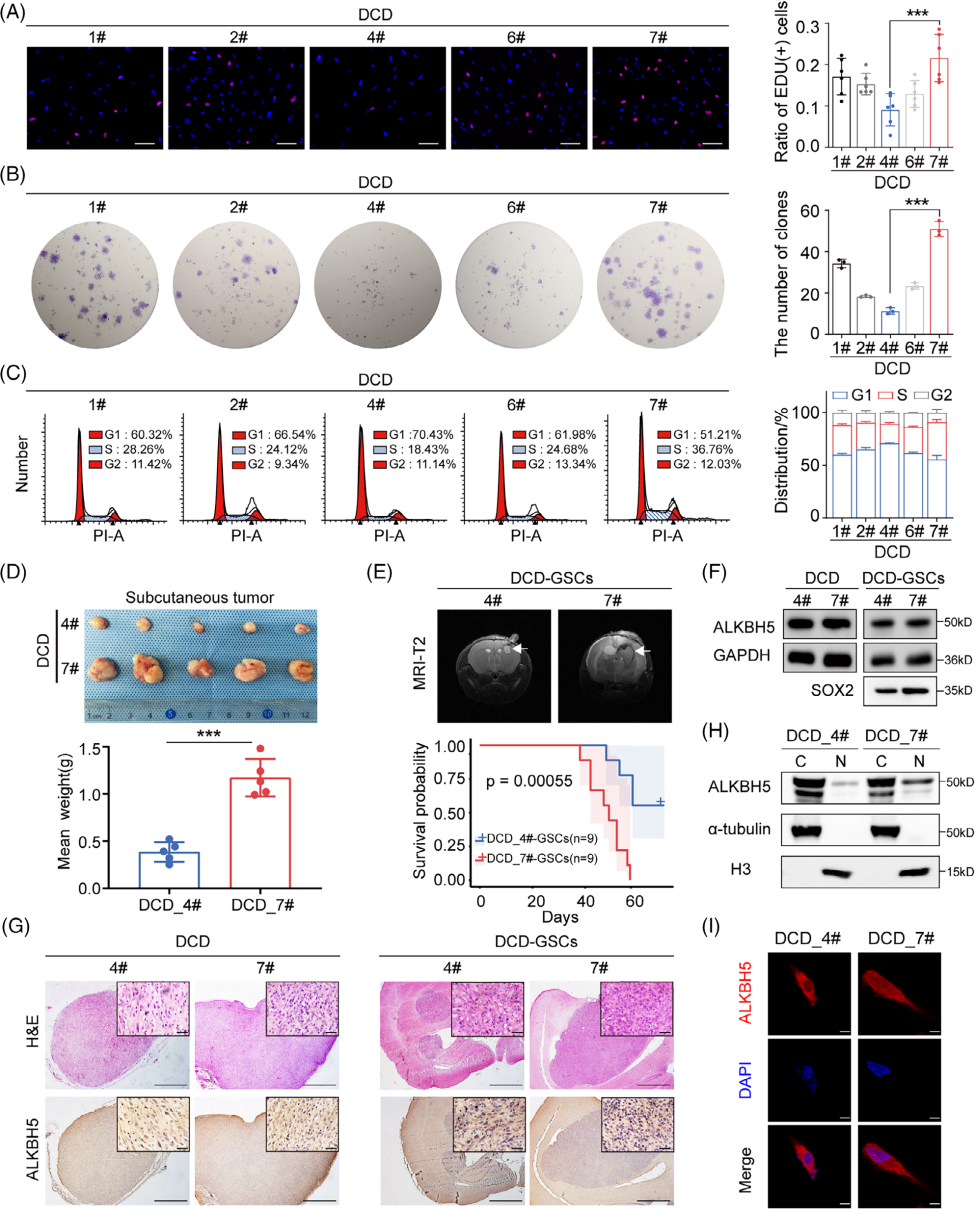
(A) EdU assay showing different cell proliferation rates in primary monoclonal cells. Scale bars = 100 µm. (B) Plate cloning assay in primary monoclonal cells. (C) Cell cycle flow analysis in primary monoclonal cells. (D) Top: Images of subcutaneous xenograft tumors in the DCD_4# and DCD_7# groups. Bottom: The mean weights of xenograft tumors in the DCD_4# and DCD_7# groups. (E) Top: Cranial MRI T2 sequence images of intracranial representative tumor‐bearing mice 3 weeks after transplantation for DCD_4# GSCs and DCD_7# GSCs cells. Bottom: Survival curves of intracranial tumor‐bearing mice in the DCD_4# GSCs and DCD_7# GSCs groups. (F) Western blot (WB) analysis of ALKBH5 and glyceraldehyde‐3‐phosphate dehydrogenase (GAPDH) in DCD_4# and DCD_7#, DCD_4# GSCs and DCD_7# GSCs; WB analysis of SOX2 in DCD_4# GSCs and DCD_7# GSCs. (G) Left: Hematoxylin and eosin (H&E) and immunohistochemistry (IHC) staining images of representative subcutaneous and intracranial tumor‐bearing mice from the DCD_4# and DCD_7#, DCD_4# GSCs and DCD_7# GSCs groups. Right: Comparison of ALKBH5 IHC staining scores among subcutaneous and intracranial tumor samples. Scale bars = 50 µm and 200 µm. (H) Nuclear/cytoplasmic fractionation assay assessing ALKBH5 in DCD_4# and DCD_7# cells. (I) Representative immunofluorescence (IF) staining showing the nuclear localization of ALKBH5 in DCD_4# and DCD_7# cells. Scale bars = 10 µm. Data are expressed as the mean ± SD. ***p < 0.001.

To investigate the ITH of GBM, we cultured GBM monoclonal cells that were derived from the same patient (NFHDCD). Importantly, these five monoclonal cells and their cancer stem‐like cells exhibited large morphological differences (Figure S2A). Furthermore, these monoclonal cells differed notably in specific functional characteristics, particularly cell vitality and the cell cycle (Figures 1A–C and S2B). DCD_4# cells had the slowest proliferation rate, while the DCD_7# cells had the fastest proliferation rate (Figures 1A–C and S2B). Compared to DCD_4# GSCs, DCD_7# GSCs had a higher glioblastoma stem‐like cells (GSC) self‐renewal capability (Figure S2C). Additionally, we analyzed tumor growth or tumor development in immunodeficient mice with intracranial tumors derived from DCD_4# and DCD_7# cells. The tumor growth derived from DCD_7# cells was larger than the tumor size derived from DCD_4# cells. Additionally, mice with intracranial tumors from the DCD_7# GSCs group had inferior survival rates when compared to the DCD_4# GSCs group (Figure 1D,E). The diverse malignant phenotype encompasses cells with varied proliferative capabilities, among which DCD_7# cells demonstrate the highest growth rate. Interestingly, despite the cells stimulating different levels of heterogeneity, ALKBH5, as a negative GBM prognostic factor that has previously been identified, did not show any significant differential expression between the GBM monoclonal cells (Figures 1F,G and S2D,E). Intriguingly, the nuclear/cytoplasmic fractionation assay and immunofluorescence (IF) assay revealed that the nuclear expression of ALKBH5 was higher in DCD_7 cells relativ e to DCD_4 cells (Figure 1H,I). The above results suggest that there is a difference in the ratio of nuclear/cytoplasm expression rather than the overall expression of ALKBH5 between monoclonal cells with significant differences in proliferative and tumorigenic capacities.

### 2.3 TDP‐43 binds to ALKBH5 via the RRM1 domain in fast‐growing GBM cells

**FIGURE 2:**
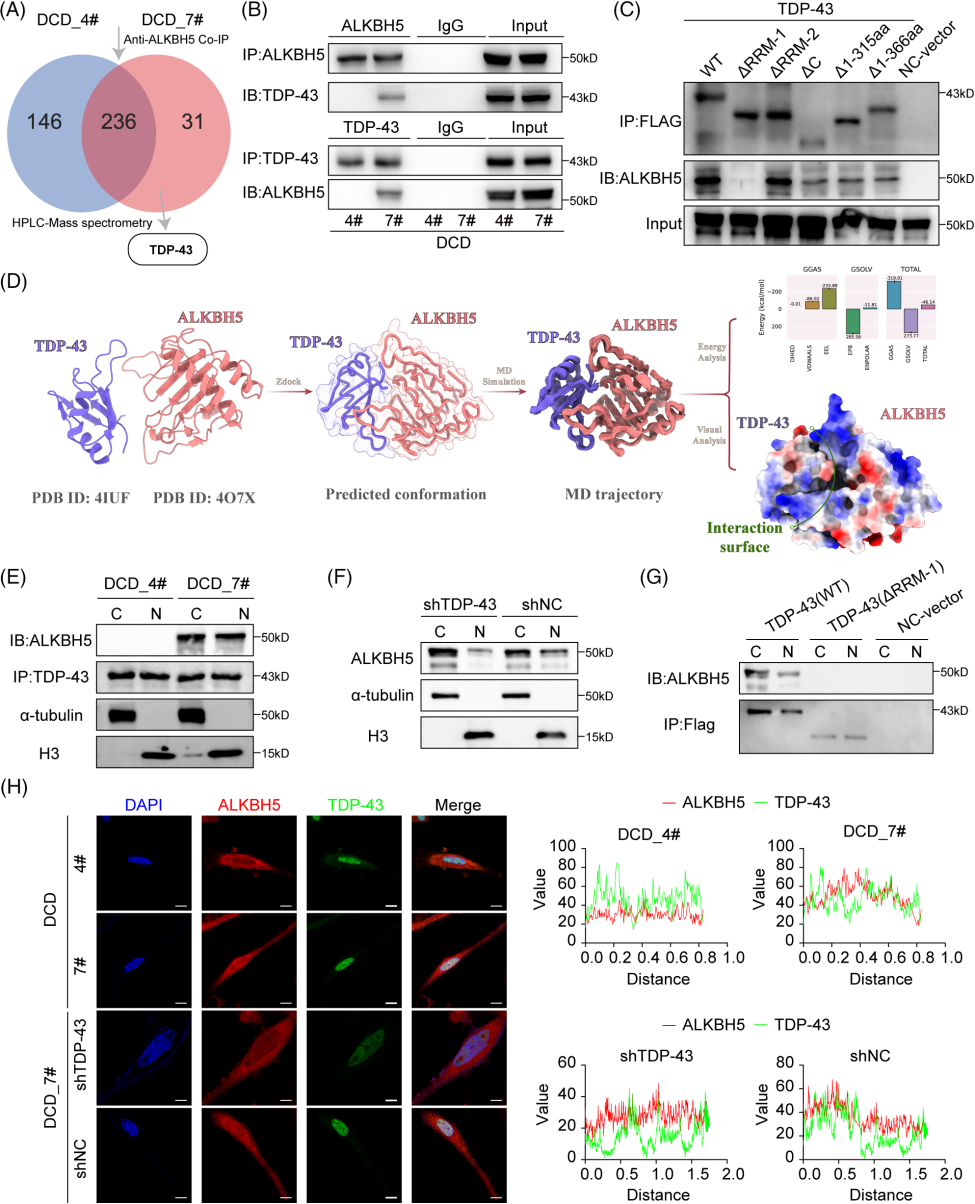
(A) Schematic of screening ALKBH5‐interacting proteins in DCD_7# cells. (B) Anti‐ALKBH5 and anti‐TAR DNA‐binding protein 43 (TDP‐43) Co‐immunoprecipitation (Co‐IP) in DCD_4# and DCD_7# cells. (C) Co‐IP assay of TDP‐43 wild type (WT) and TDP‐43 truncates. (D) Schematic of TDP‐43 and ALKBH5 complex analysis. (E) Nuclear/cytoplasmic fractionation followed by immunoprecipitation (IP) assay assessing the binding of TDP‐43 and ALKBH5 in DCD_4# cells and DCD_7# cells. (F) Nuclear/cytoplasmic fractionation assay assessing ALKBH5 in DCD_7# cells after TDP‐43 depletion. (G) Nuclear/cytoplasmic fractionation followed by IP assay assessing the binding of TDP‐43 and ALKBH5 in DCD_7# cells after transfecting TDP‐43(WT) and TDP‐43(ΔRRM1). H Representative immunofluorescence (IF) staining showing the nuclear localization of ALKBH5 and TDP‐43 in DCD_4# and DCD_7# cells, DCD_7# cells after TDP‐43 depletion. Scale bars = 10 µm.

Protein–protein interactions play a crucial role in regulating tumor progression,^35^ with RNA‐binding proteins frequently assuming a key role in post‐transcriptional modifications via intricate interactions.^36^ Prompted by the above results, we further explored the interacting proteins of ALKBH5 and their potential mechanisms. Thus, we used anti‐ALKBH5 Co‐immunoprecipitation (Co‐IP) following high‐performance liquid chromatography (HPLC)–mass spectrometry analysis to compare the differential mutually binding proteins of ALKBH5 (Figure 2A). We selected 31 proteins that differentially combined with ALKBH5 by at least twofold in DCD_7# cells for Gene Ontology (GO) and STRING analysis. We found that the identified ALKBH5‐binding proteins were primarily enriched for “RNA splicing” and “regulation of the cell cycle” terms based on GO analysis (Figure S3A). Among these proteins, TDP‐43 was considered a key protein due to its critical position in the protein‒protein interaction (PPI) network (Figure S3B) and its essential role in inhibiting RNA splicing.^37^, ^38^ Anti‐ALKBH5 Co‐IP assays indicated that TDP‐43 exclusively bound to ALKBH5 in DCD_7# cells but not in DCD_4# cells (Figure 2B). Furthermore, we found that ALKBH5 specifically binds to TDP‐43 in DCD7# cells following anti‐TDP‐43 Co‐IP (Figure 2B). We constructed five TDP‐43 mutants (TDP‐43 ΔRRM1, TDP‐43 ΔRRM2, TDP‐43 ΔC, TDP‐43 Δ1‐315aa, and TDP‐43 Δ1‐366aa) with FLAG‐tag to evaluate their ability to bind ALKBH5 through Co‐IP in DCD_7# cells (Figure S3C). We found that TDP‐43 ΔRRM2, TDP‐43 Δ1‐366aa, TDP‐43 Δ1‐315aa, and TDP‐43 ΔC could all bind ALKBH5 similarly to TDP‐43 wild type (WT), but TDP‐43 ΔRRM1 could not (Figure 2C). These results suggest that the TDP‐43 RRM1 domain is necessary for its interaction with ALKBH5.Given that the crystal structure of the TDP‐43/ALKBH5 complex has not been reported in the literature, our study aimed to delve deeper into the interactions between the two proteins (Figure 2D). We initially employed molecular docking techniques to predict the potential binding modes of TDP‐43 and ALKBH5, uploading their structural data to the Zdock online server (http://zdock.umassmed.edu/) for protein–protein docking simulations (Figure 2D). After comparing the predicted results, we selected the highest‐scoring binding model for subsequent optimization (Figure 2D). We carried out a 100 ns molecular dynamics simulation using the Gromacs 2022.3 software, hoping to glean insights into the binding characteristics from the simulation trajectories and binding free energy analyses. Upon completion of the simulation, we calculated the root‐mean‐square deviation (RMSD) for both the ALKBH5/TDP‐43 complex and the individual monomers and found that TDP‐43 and ALKBH5 maintained structural stability after 60 ns (Figure S3D). Based on these observations, we decided to sample the stable trajectories from 60 ns to 100 ns for in‐depth analysis. Furthermore, we computed and analyzed the root‐mean‐square fluctuation (RMSF) and B‐factors for TDP‐43 and ALKBH5 during this time frame (Figure S3E). The RMSF values for most amino acid residues remained stable, with values below 0.2 nm (Figure 2F). Notably, there were regions within ALKBH5 that exhibited higher RMSF values, indicating increased flexibility (Figure S3E). It is worth mentioning that these areas with higher RMSF did not participate in direct interaction with TDP‐43, while the amino acid residues directly involved in the interaction displayed relative stability, further corroborating the positive contribution of the ALKBH5and TDP‐43 binding to the overall protein stability (Figure S3E). Subsequently, we analyzed the molecular dynamics simulation trajectories to enumerate the number of hydrogen bonds formed between TDP‐43 and ALKBH5. There was a progressive increase in the number of hydrogen bonds, indicating a strengthening of the binding affinity between TDP‐43 and ALKBH5 (Figure S3F). Further, we extracted the final frame from the simulation trajectories to conduct a detailed visual analysis of the interaction patterns between TDP‐43 and ALKBH5. An extensive network of hydrogen bonds at the interaction interface between ALKBH5 and TDP‐43, contributing significantly to the stabilization of their complex (Figure S3G). The stable association between proteins depends not only on the complementarity and interactions of their structures but also critically on the interplay of electrostatic potentials. In our study, we utilized ChimeraX to calculate and visualize the distribution of electrostatic potentials between ALKBH5 and TDP‐43. As demonstrated in the corresponding images, at the protein–protein interaction interface, the negative potential regions of ALKBH5 (depicted in blue) and the positive potential regions of TDP‐43 (depicted in red) exhibit a pronounced complementarity (Figure S3H). This electrostatic complementarity not only reveals the interaction characteristics between the two proteins but also provides substantial molecular evidence for the enhanced stability of the ALKBH5‐TDP‐43 association (Figure S3H). Moreover, we utilized the gmx_MMPBSA tool to perform an in‐depth analysis of the binding free energy between TDP‐43 and ALKBH5 proteins. A total of 1000 frames were extracted from the last 10 ns of molecular dynamics trajectories to calculate the binding free energy between TDP‐43 and ALKBH5. The average binding free energy was maintained at approximately −46.14 kcal/mol, with the gas‐phase free energy (GGAS) contributing −319.91 kcal/mol and the solvation free energy (GSOLV) contributing 273.77 kcal/mol (Figure S3I,J). Further decomposition of the binding free energy into the contributions from individual amino acids at the interaction interface revealed significant energetic contributions from certain residues. Specifically, in TDP‐43, Leu109, Leu111, Trp113, Leu139, Lys140, and Phe147, and in ALKBH5, His122, Arg130, Leu145, Gln146, Cys200, and Val202 residues played pivotal roles in the binding, with some of these amino acids involved in the formation of hydrogen bonds at the interaction interface (Figure S3K,L). Intriguingly, TDP‐43 domain contained exactly the majority of the above predicted residues.

We utilized DCD_4# cells and DCD_7# cells for nuclear/cytoplasmic fractionation followed by immunoprecipitation (IP) experiments, which showed that TDP‐43 and ALKBH5 were bound in both the cytoplasm and nucleus of DCD_7# cells (Figure 2E). The nuclear translocation of ALKBH5 was hindered when TDP‐43 expression was disrupted in DCD_7# cells (Figure 2F). Significantly, the transfection of TDP‐43 ΔRRM1 mutants in DCD_7# cells inhibited the interaction between TDP‐43 and ALKBH5, resulting in the subsequent suppression of ALKBH5 translocation into the nucleus (Figure 2G). Using confocal microscopy analysis, we observed significantly more fluorescent staining of ALKBH5 and TDP‐43 in the nucleus in DCD_7# cells than in DCD_4# cells (Figure 2H). Notably, silencing TDP‐43 reduced the nuclear localization of ALKBH5 in DCD_7# cells (Figure 2H). The above results show that TDP‐43 binds to ALKBH5 through its RRM1 structural domain and promotes its nuclear translocation.

### 2.4 TDP‐43 inhibition decreases the proliferation and tumorigenesis of GBM

**FIGURE 3:**
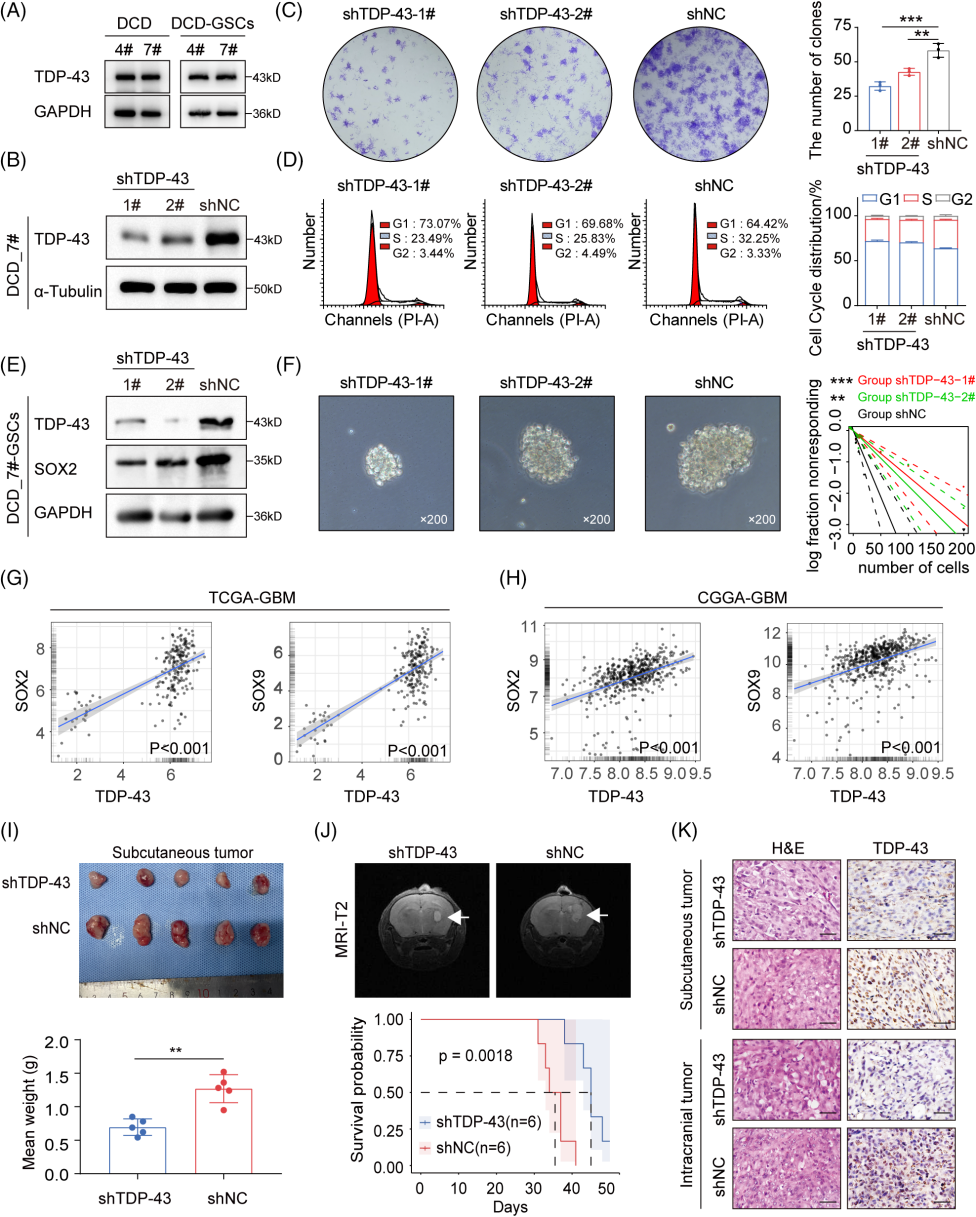
(A) Western blot (WB) analysis of TAR DNA‐binding protein 43 (TDP‐43) and glyceraldehyde‐3‐phosphate dehydrogenase (GAPDH) in DCD_4# and DCD_7# cells and DCD_4# and DCD_7# GSCs. (B) WB analysis of TDP‐43 and α‐Tubulin in DCD_7# cells after TDP‐43 depletion. (C) Plate cloning assay in DCD_7# cells after the indicated shRNA transfection. (D) Cell cycle flow analysis in DCD_7# cells after the indicated shRNA transfection. (E) WB analysis of ALKBH5, SOX2 and GAPDH in DCD_7# GSCs after TDP‐43 depletion. (F) Extreme limiting dilution analysis (ELDA) in DCD_7# GSCs after TDP‐43 depletion. (G, H) The Pearson correlation between TDP‐43 and SOX2, TDP‐43 and SOX9 mRNA expression in the TCGA and Chinese Glioma Genome Atlas (CGGA) glioblastoma (GBM) dataset. (I) Top: Images of subcutaneous xenograft tumors in the shTDP‐43 and shNC groups. Bottom: The mean weights of xenograft tumors in the shTDP‐43 and shNC groups. (J) Top: Representative cranial MRI T2 sequence images of intracranial tumor‐bearing mice 3 weeks after transplantation of shTDP‐43 and shNC cells. Bottom: Survival curves of intracranial tumor‐bearing mice in the shTDP‐43 and shNC groups. (K) Hematoxylin and eosin (H&E) and immunohistochemistry (IHC) staining images of representative subcutaneous and intracranial tumor‐bearing mice from the shTDP‐43 and shNC groups. Scale bars = 50 µm. Data are expressed as the mean ± SD. **p < 0.01; ***p < 0.001.

There was also no statistical difference in overall protein expression of TDP‐43 between DCD_4# and DCD_7# cells or their GSCs (Figure 3A). In DCD_7# and U87MG cells, TDP‐43 expression was diminished using siRNAs, which resulted in a significant decrease in cell growth across all cell lines (Figure S4A–E). Targeting TDP‐43 expression in DCD_7# cells with shRNA lentiviral transfection inhibited the clone‐forming capacity and hindered the G1/S phase transition (Figure 3B–D). Notably, loss of TDP‐43 substantially reduced stemness properties (Figure 3E,F). Analyses of TCGA and CGGA GBM data revealed a positive correlation between TDP‐43 mRNA expression and SOX2, as well as SOX9 (Figure 3G,H).

We conducted Kaplan–Meier survival curves on NFH survival data to determine the correlation between TDP‐43 expression (according to IHC data) and overall survival (OS) in GBM patients (Figure S4F,G and Table S2). The results revealed that TDP‐43 indicates an unfavorable GBM prognosis (Figure S4G). We evaluated the impact of TDP‐43 depletion on tumorigenicity by subcutaneous and intracranial injections into nude mice with DCD_7# cells transduced with shNC or shTDP‐43. Compared to the mice that received shNC DCD_7# cells, those that received shTDP‐43 DCD_7# cells exhibited prolonged survival with a lower rate of tumor formation (Figure 3I,J). The efficiency of TDP‐43 interference in situ was verified using an IHC assay (Figure 3K). These findings collectively suggest that TDP‐43 promotes the proliferation and tumorigenicity of GBM cells.

### 2.5 Analysis of m^6^A targets in fast‐growing GBM cells

**FIGURE 4:**
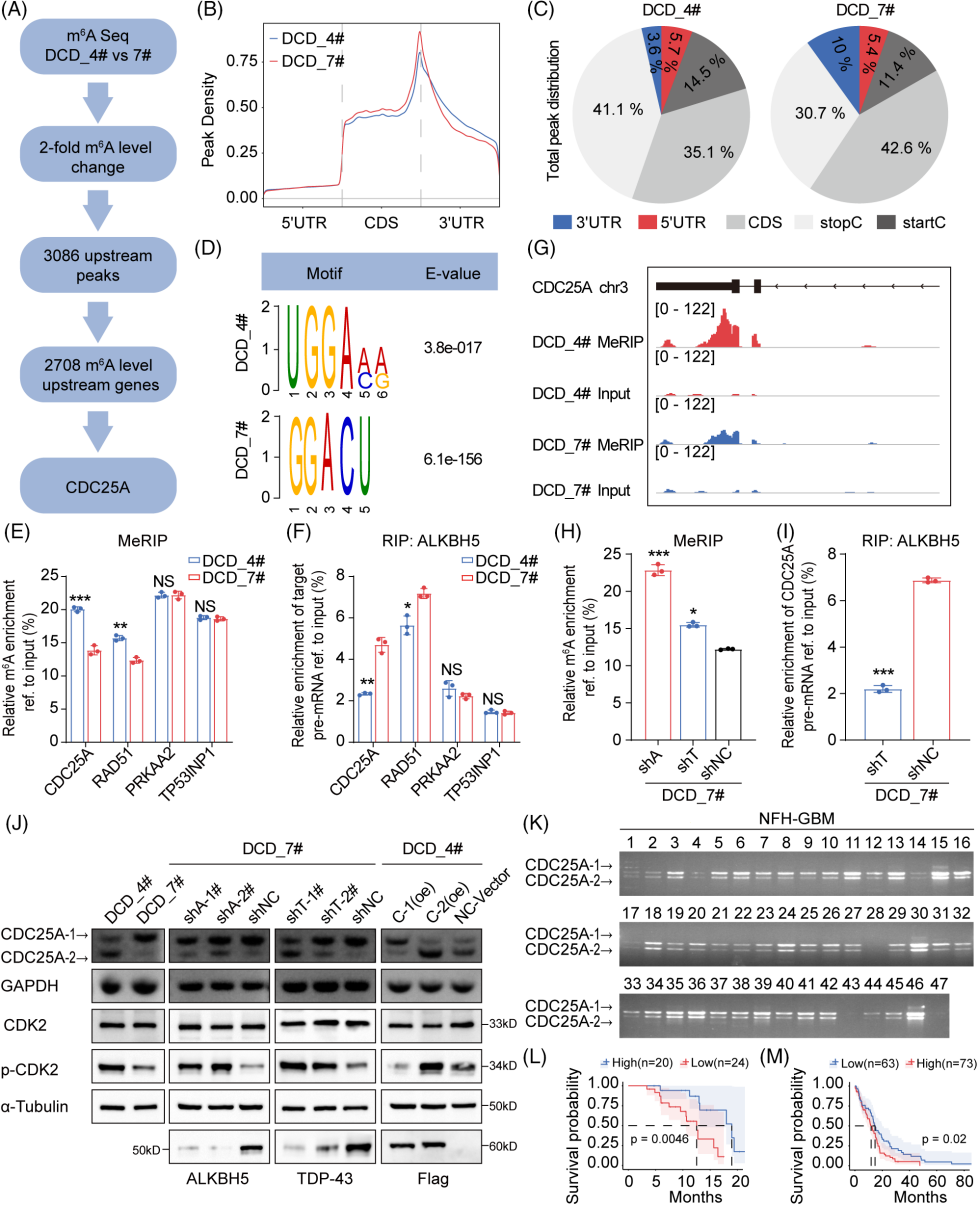
(A) Schematic of screening N6‐methyladenosine (m^6^A) methylation upregulated genes in DCD_4# cells. (B) Peak density in MeRIP‐seq results. (C) Total peak distribution of m^6^A modification in MeRIP‐seq results. (D) The RRACH motif identified from MeRIP‐seq peaks in DCD_4# and DCD_7# cells. (E) MeRIP‐qPCR analysis of m^6^A levels ofcell division cycle 25A (CDC25A), RAD51, PRKAA2, and TP53INP1 in DCD_4# and DCD_7# cells. (F) RIP‐qPCR analysis of the enrichment of CDC25A, RAD51, PRKAA2, and TP53INP1 on ALKBH5 in DCD_4# and DCD_7# cells. (G) m^6^A abundances in CDC25A mRNA transcripts in DCD_4# and DCD_7# cells. (H) MeRIP‐qPCR analysis of m^6^A levels of CDC25A pre‐mRNA in DCD_7# cells after ALKBH5 or TAR DNA‐binding protein 43 (TDP‐43) depletion. (I) RIP‐qPCR analysis of the enrichment of CDC25A pre‐mRNA on ALKBH5 in DCD_7# cells after TDP‐43 depletion. (J) Top: Agarose electrophoretogram analysis of the isoform switch of CDC25A in DCD_4# and DCD_7# cells, DCD_7# cells after ALKBH5 or TDP‐43 depletion and DCD_4# cells after CDC25A‐1(oe) and CDC25A‐2(oe) transfection. Bottom: Western blot (WB) analysis of CDK2, p‐CDK2 and α‐Tubulin in DCD_4# and DCD_7# cells, DCD_7# cells after ALKBH5 or TDP‐43 depletion and DCD_4# cells after CDC25A‐1(oe) and CDC25A‐2(oe) transfection. (K) The agarose electrophoretogram analysis for the isoform switch of CDC25A in NFH glioblastoma (GBM) samples. (L) Kaplan–Meier survival curve of NFH‐GBM patients stratified by CDC25A‐2/CDC25A‐1 level (according to agarose electrophoretogram data). Kaplan–Meier survival curves of GBM patients in TCGA datasets stratified by the percentage of spliced‐in index (PSI) value of CDC25A exon 6. (M) Data are expressed as the mean ± SD. NS, no significance; *p < 0.05; **p < 0.01; ***p < 0.001.

To further explore the demethylation function of ALKBH5 after its interaction with TDP‐43, we employed MeRIP‐seq and identified 3086 upstream m^6^A peaks and 2708 genes with at least a twofold m^6^A level increase in DCD_4# cells compared to DCD_7# cells (Figure 4A). Our analysis revealed that the m^6^A signal occurred primarily near the stop codon and the 3′ UTR of the mRNA transcript (Figure 4B). Additionally, we analyzed the distribution patterns of the m^6^A peaks within the total peaks (Figure 4C) and found that the RRACH motif was highly enriched within the m^6^A sites in DCD_4# and DCD_7# cells (Figure 4D). RRACH (R = A or G, H = A, C, or U) has been reported to be a conserved sequence for m^6^A modification.^26^ The m^6^A‐modified genes were mainly enriched for the “cell cycle” and “cell cycle phase transition” terms according to our GO and KEGG analyses (Figure S5A,B). We identified “m^6^A hypomethylation” and “cell cycle‐related” genes, such as CDC25A, RAD51, PRKAA2, and TP53INP1, as candidate genes in DCD‐7# cells. These genes were validated in DCD_4# and DCD_7# cells through MeRIP and RIP experiments, revealing CDC25A as the most statistically different among all candidate genes (Figure 4E,F). Notably, we observed a significant increase in m^6^A abundance in the 5′ untranslated regions (5′ UTRs) of CDC25A mRNA (chr3:48,196,558–48,231,691) in DCD_4# cells compared to DCD_7# cells (Figure 4G). Therefore, we selected CDC25A (transcript ID: NM_001789.3), an essential regulator of G1/S phase, for further analysis.

### 2.6 TDP‐43/ALKBH5 regulates CDC25A mRNA splicing via m6A demethylation

Moreover, the m^6^A levels of fragments corresponding to the m^6^A modification sites were substantially increased upon ALKBH5 or TDP‐43 depletion in DCD_7# cells (Figure 4H). The RIP assays demonstrated that ALKBH5 preferably binds CDC25A pre‐mRNA in DCD_7# cells compared to DCD_4# cells (Figure 4F). However, this binding was disrupted after the knockdown of TDP‐43 in DCD_7# cells (Figure 4I). Additionally, we observed the isoform switch of CDC25A from the full‐length isoform CDC25A‐1 (transcript ID: NM_001789.3) to the truncated isoform CDC25A‐2 (transcript ID: NM_201567.2), a reported alternative splicing event in teratocarcinoma not previously studied in the context of GBM^39^ (Figure 4J). Knockdown of ALKBH5 or TDP‐43 expression promoted exon 6 skipping of CDC25A (Figure 4J), impacting its entry into the G1 phase and the G1/S transition by dephosphorylating p‐T14, an inhibitory phosphorylation residue on CDK2.^39^ The expression of p‐CDK2 was significantly higher in DCD_4# cells compared to DCD_7# cells (Figure 4J). Subsequent knockdown of ALKBH5 or TDP‐43 expression in DCD_7# cells further elevated the expression of p‐CDK2 (Figure 4J). Following the transfection of CDC25A‐1(oe) and CDC25A‐2(oe) in DCD_4# cells, the ratio of CDC25A‐1 to CDC25A‐2 was modified, consequently impacting p‐CDK2 expression (Figure 4J). Additionally, lower levels of CDC25A‐2/CDC25A‐1 were indicative of poor prognosis for GBM patients operated on at NFH (Figure 4K,L). Furthermore, the percentage of spliced‐in index (PSI) values of CDC25A exon 6 showed a negative association with OS for GBM (Figure 4M). These results suggest that ALKBH5 maintains the expression of the oncogenic isoform CDC25A‐1 via m^6^A demethylation.

### 2.7 TDP‐43/ALKBH5‐mediated expression of oncogenic isoform CDC25A‐1 promotes the growth of fast‐growing GBM cells

**FIGURE 5:**
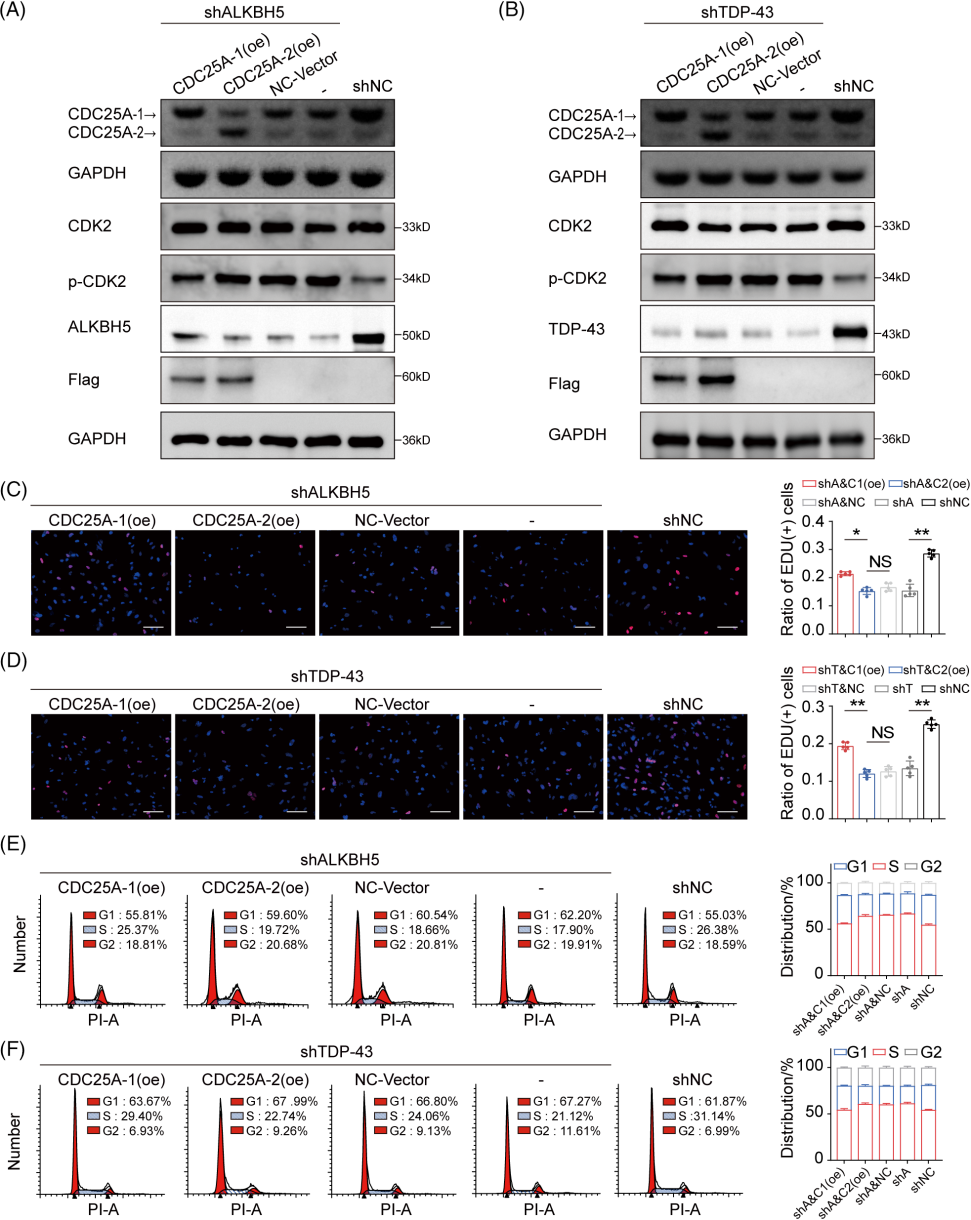
(A) Agarose electrophoretogram and Western blot (WB) analysis of shALKBH5 cells overexpressing cell division cycle 25A‐1 (CDC25A‐1) or CDC25A‐2. (B) Agarose electrophoretogram and WB analysis of shTDP‐43 cells overexpressing CDC25A‐1 or CDC25A‐2. (C) Recovery EdU assays in shALKBH5 cells overexpressing CDC25A‐1 or CDC25A‐2. Scale bars = 100 µm. (D) Recovery EdU assays in shTDP‐43 cells overexpressing CDC25A‐1 or CDC25A‐2. Scale bars = 100 µm. (E) Cell cycle flow analysis in shALKBH5 cells overexpressing CDC25A‐1 or CDC25A‐2. (F) Cell cycle flow analysis in shTDP‐43 cells overexpressing CDC25A‐1 or CDC25A‐2. Data are expressed as the mean ± SD. NS, no significance; *p < 0.05; **p < 0.01.

The knockdown of ALKBH5 or TDP‐43 expression led to a reduction in the oncogenic isoform CDC25A‐1 in DCD_7# cells (Figure 5A,B). Subsequent transfection with CDC25A‐1(oe) and CDC25A‐2(oe) plasmids showed that overexpression of CDC25A‐1 caused partial recovery of the heightened level of CDC25A‐2/CDC25A‐1, whereas exogenous CDC25A‐2 exacerbated the level of CDC25A‐2/CDC25A‐1 (Figure 5A,B). Moreover, overexpression of CDC25A‐1 alleviated the inhibitory effect of the phosphorylation site on CDK2 (Figure 5A,B). Importantly, we observed similar rescues in proliferation inhibition and G1/S phase block caused by isoform switching of CDC25A (Figures 5C–F). These results further support the conclusion that CDC25A mRNA splicing is important for G1/S phase transition and proliferative phenotype in fast‐growing GBM cells.

### 2.8 TDP‐43 RRM1 domain is required for the isoform switch of CDC25A

**FIGURE 6:**
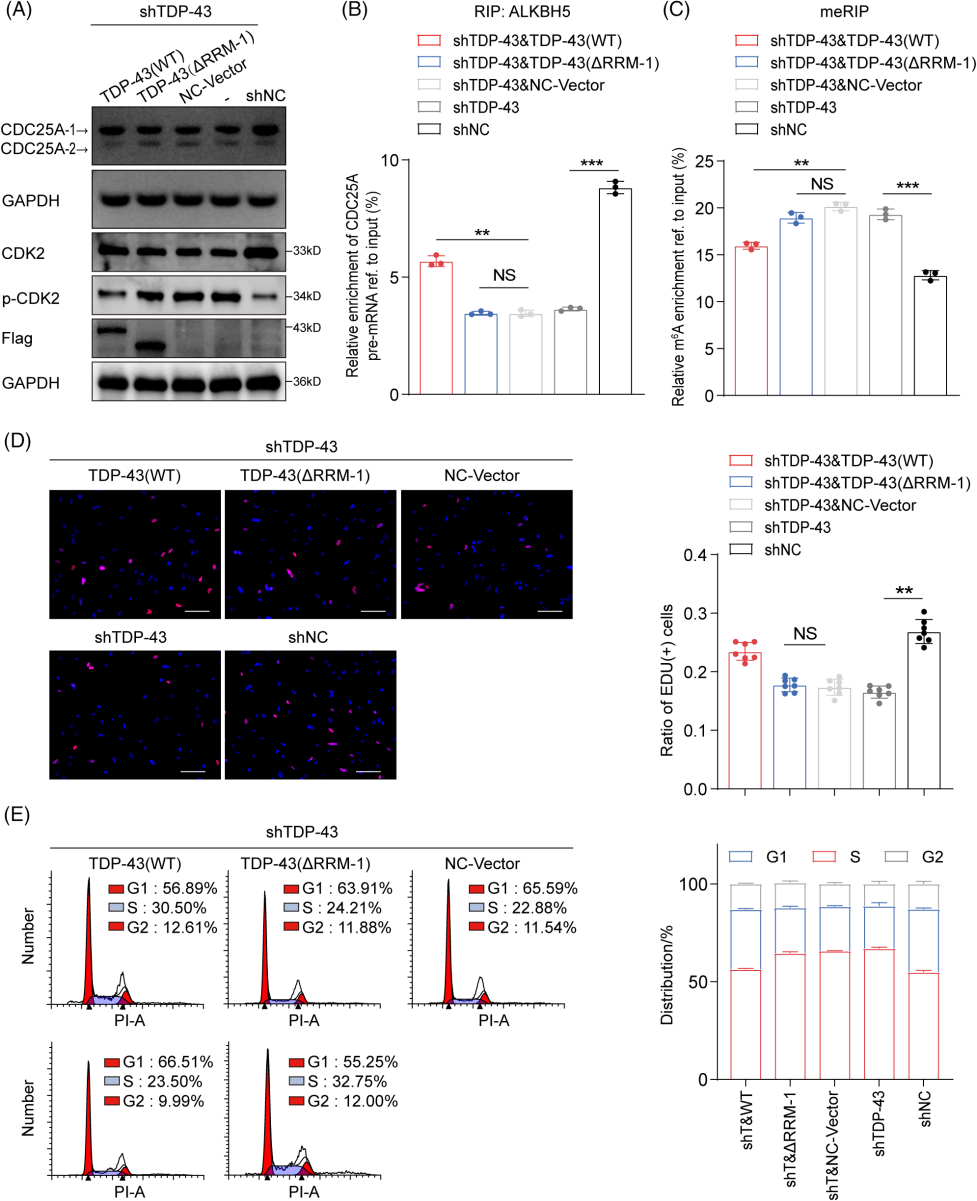
(A) Agarose electrophoretogram and Western blot (WB) analysis of shTDP‐43 cells after TAR DNA‐binding protein 43 (TDP‐43) wild type (WT) or TDP‐43 ΔRRM1 transfection. (B) RIP‐qPCR analysis of the enrichment of cell division cycle 25A (CDC25A) pre‐mRNA on ALKBH5 in shTDP‐43 cells after TDP‐43 WT or TDP‐43 ΔRRM1 transfection. (C) MeRIP‐qPCR analysis of N6‐methyladenosine (m^6^A) levels of CDC25A pre‐mRNA in DCD_7# cells after TDP‐43 depletion. (D) Recovery EdU assay in shTDP‐43 cells after TDP‐43 WT or TDP‐43 ΔRRM1 transfection. Scale bars = 100 µm. (E) Cell cycle flow analysis in shTDP‐43 cells after TDP‐43 WT or TDP‐43 ΔRRM1 transfection. Data are expressed as the mean ± SD. NS, no significance; **p < 0.01; ***p < 0.001.

To demonstrate that CDC25A exon 6 skipping is associated with the binding of TDP‐43 and ALKBH5, we conducted restoration experiments using WT and mutant forms of TDP‐43. Transfection of TDP‐43 WT significantly restored the expression of CDC25A‐1 levels while partially reversing the upregulation of CDC25A‐2 levels (Figure 6A). In contrast, mutant TDP‐43 lacking RRM1 failed to restore CDC25A‐1 levels (Figure 6A). In addition, transfection of TDP‐43 WT, but not TDP‐43 ΔRRM1, partially alleviated the inhibitory effect of the phosphorylation site on CDK2 in shTDP‐43 DCD_7# cells (Figure 6A). TDP‐43 ΔRRM1 transfection failed to restore ALKBH5 binding to CDC25A and did not cause the same reduction in CDC25A m^6^A levels as TDP‐43 WT transfection (Figure 6B,C). Importantly, we found that cell growth inhibition and G1/S phase arrest caused by CDC25A exon 6 skipping were similarly abolished by retransfection with TDP‐43 WT but not TDP‐43 ΔRRM1 (Figure 6D,E). Therefore, it can be concluded that the presence and functionality of the TDP‐43 RRM1 domain is crucial for the regulation of the isoform switch of CDC25A.

### 2.9 Targeting the TDP‐43 and ALKBH5 interaction is a feasible treatment for GBM

**FIGURE 7:**
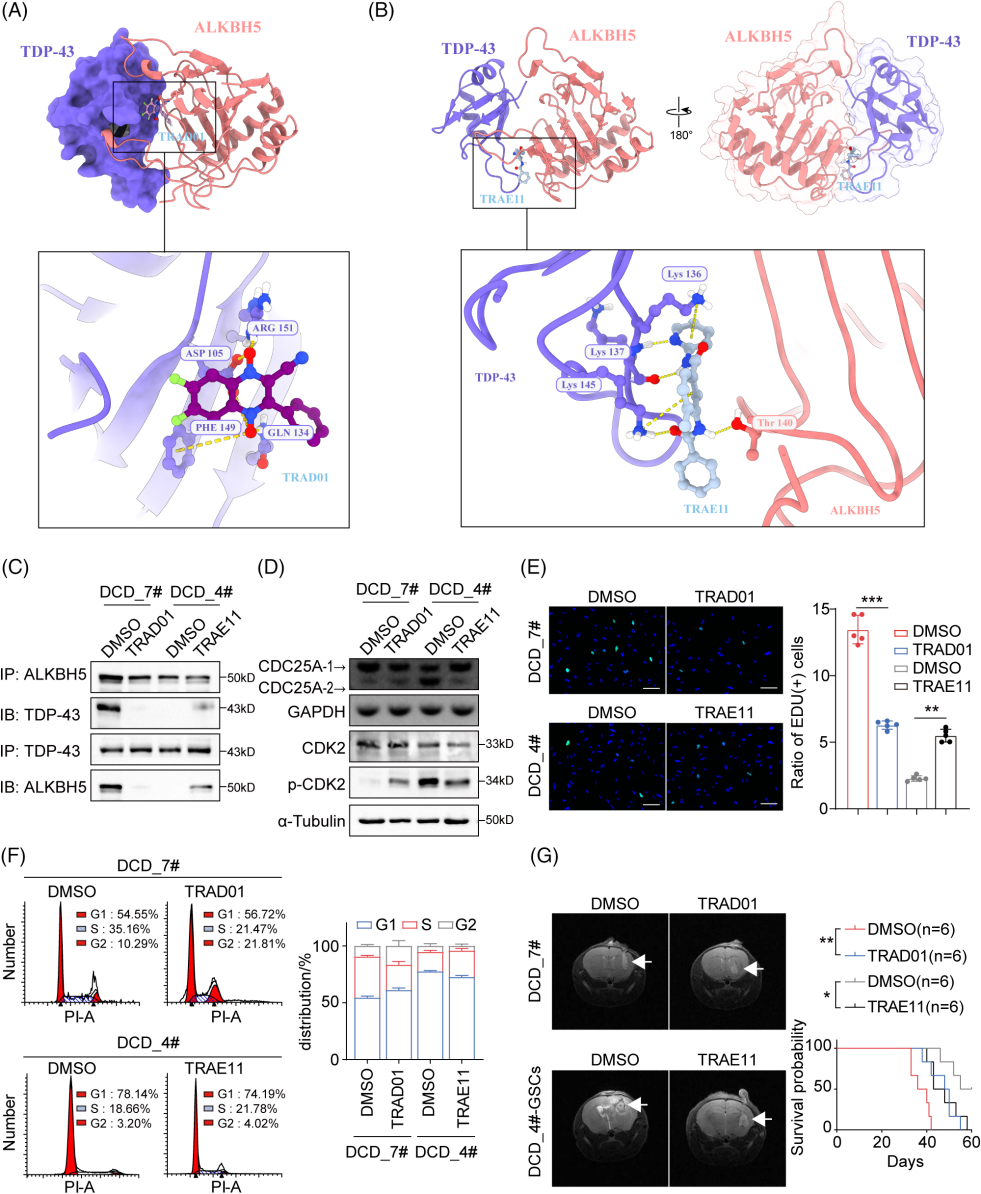
(A) The binding mode of TRAD01 and TAR DNA‐binding protein 43 (TDP‐43)‐ALKBH5 complex. (B) The binding mode of TRAE11 and TDP‐43‐ALKBH5 complex. (C) Anti‐ALKBH5 and anti‐TDP‐43 Co‐immunoprecipitation (Co‐IP) in DCD_7# cells after TRAD01 treatment (300 nmol/L) and DCD_4# cells after TRAE11 treatment (800 nmol/L). (D) Top: Agarose electrophoretogram analysis of the isoform switch of cell division cycle 25A (CDC25A) in DCD_7# cells after TRAD01 treatment and DCD_4# cells after TRAE11 treatment. Bottom: Western blot (WB) analysis of CDK2, p‐CDK2 and α‐Tubulin in DCD_7# cells after TRAD01 treatment and DCD_4# cells after TRAE11 treatment. (E) EdU assay showing different cell proliferation rates in DCD_7# cells after TRAD01 treatment and DCD_4# cells after TRAE11 treatment. Scale bars = 100 µm. (F) Cell cycle flow analysis in DCD_7# cells after TRAD01 treatment and DCD_4# cells after TRAE11 treatment. (G) Left: Cranial MRI T2 sequence images of intracranial representative tumor‐bearing mice 3 weeks after transplantation for DCD_7# cells after TRAD01 treatment and DCD_4# cells after TRAE11 treatment. Right: Survival curves of intracranial tumor‐bearing mice in DCD_7# cells after TRAD01 treatment and DCD_4# cells after TRAE11 treatment. Data are expressed as the mean ± SD. *p < 0.05; **p < 0.01; ***p < 0.001.

Since the ChemDiv database was the most diverse and pharmacologically relevant, a virtual screen was performed using the ChemDiv database to identify small molecules with the ability to inhibit or enhance the formation of the TDP‐43/ALKBH5 complex. The resulting docking poses were analyzed based on a docking score, and the swissADME database was utilized to predict the pharmacokinetics of small molecule compounds for subsequent screening. We selected the inhibitor with the best TOP docking score and predicted blood–brain barrier permeant by SwissADME, N#Cc1c(‐c2ccccc2)[n+]([O‐])c(cc(c(F)c2)F)c2[n+]1[O‐], hereafter referred to as TRAD01 for TDP‐43 RRM1‐ALKBH5 disruptor 01 (Figure S6A). It produces hydrogen bonding and salt‐bridge interactions with ASP105, GLN134, PHE149 and ARG151 of TDP‐43 (Figure S6B), thus hindering the binding of TDP‐43 to ALKBH5, as shown by the crystal structure (Figure 7A). Next, We used a similar strategy to screen for the enhancer, n1ccccc1NC(=O)c2cc(c©cc2)NC(=O)c3ccccc3, hereafter referred to as TRAE11 for TDP‐43 RRM1‐ALKBH5 enhancer 11(Figure S6C). TRAE11 binds to the interacting surface of TDP‐43 and ALKBH5, facilitating their interaction and promoting their binding (Figures 7B and S6D). To investigate the potential therapeutic value of the small molecule TRAD01 in GBM, we measured its inhibitory concentration (IC_50_) in DCD7# cells (Figure S6E) and verified its ability to effectively block the binding of ALKBH5 and TDP‐43 via anti‐ALKBH5 and anti‐TDP‐43 Co‐immunoprecipitation (Co‐IP) in TRAD01‐treated DCD_7# cells (Figure 7C). Additionally, the Co‐IP assay results demonstrated that TRAE11 enhances the interaction between TDP‐43 and ALKBH5 in DCD_4# cells (Figure 7C). TRAD01 promoted CDC25A exon 6 skipping in DCD_7# cells, leading to enhanced the inhibitory effect of the phosphorylation site on CDK2, whereas TRAE11 inhibited the isoform switch of CDC25A in DCD_4# cells, resulting in alleviated the inhibitory effect of the phosphorylation site on CDK2 (Figures 7D). In vitro experiments showed that TRAD01 effectively inhibited the proliferation of DCD_7# cells, while TRAE11 significantly promoted the growth of DCD_4# cells (Figures 7E and S6F). TRAD01 blocked the G1/S phase transition in DCD_7# cells, whereas TRAE11 promoted the G1/S phase transition in DCD_4 cells (Figures 7F). Furthermore, we observed the effect of TRAD01‐treated DCD_7# cells on the survival time of tumor‐bearing mice by injecting them into nude mice (Figures 7G). TRAE11 treatment led to reduced survival rates in mice bearing tumors that had been injected with DCD_4# GSC cells treated with TRAE11(Figures 7G). These results further validated the impact of the TDP‐43 and ALKBH5 interaction on the growth rate of GBM cells. Thus, targeting the binding of TDP‐43 and ALKBH5 with small molecules might be a promising approach to target fast‐proliferating cells in GBM.

## 3 DISCUSSION

GBM is a type of brain tumor characterized by ITH that leads to a limited response to targeted therapy.^8^, ^9^ Our study focuses on ITH and aims to tackle this clinical challenge through the study of epigenetic mechanisms. Recent studies provide strong evidence supporting the epigenetic basis of cell fate determination and ITH in GBM.^13^ Uncovering the epigenetic mechanisms behind ITH regulation can shed light on potential GBM treatment options.

ALKBH5 has been reported to play an important role in GBM. ALKBH5 deficiency decreased PD‐L1 protein level by suppressing ZDHHC3 mRNA expression in an m^6^A modification manner, further enhancing the efficacy of anti‐PD‐1 therapy for GBM.^40^ ALKBH5 facilitates hypoxia‐induced paraspeckle assembly and IL8 secretion to recruit tumor‐associated macrophages (TAMs) to promote GBM progression.^41^ Ubiquitin‐specific peptidase 36 (USP36) interacts with and stabilizes ALKBH5, and targeting USP36‐ALKBH5 axis inhibits cell proliferation and self‐renewal of GSCs and sensitized GSCs to temozolomide (TMZ) treatment.^42^ It has been reported that ALKBH5 maintains tumorigenicity of GBM stem‐like cells (GSCs) by sustaining FOXM1 mRNA expression and cell proliferation program.^26^ We also found that ALKBH5 expression was associated with malignant proliferation of GBM and stemness of GSCs, but we focused on the alternative splicing events and isoform switch of target genes rather than mRNA expression. In this study, we show that ALKBH5 regulates AS events through m^6^A demethylation thereby affecting the ITH‐mediated diverse proliferation phenotypes of GBM.

Dysregulated cell cycle progression is a hallmark of tumorigenesis, which manifests through mutations, epigenetic changes, and post‐translational modifications of cell cycle checkpoints.^12^ The cell cycle moves from the G1 to S phase through the expression of dihydrofolate reductase (DHFR), thymidine kinase (TK), and ribonucleotide reductase (RR), which are all regulated by E2F binding sites in their promoter regions.^43^ CDC25A dephosphorylates the inhibitory phosphorylation motifs in the cyclin‐dependent kinase 2 (CDK2) protein, which leads to CDK2 activation, binding to cyclin E, phosphorylation of retinoblastoma (Rb) protein, and ultimately, dissociation of Rb from E2F transcriptional activator, release of repression of E2F transcriptional activator activity, thus promoting the transcription of DNA synthesis‐related proteins and aiding the G1/S phase transition.^43^ CDC25A−/− pure and knockout mice show significant suppression of cell development, cellular degradation, and cell death early in embryonic development.^44^ Growing evidence suggests that CDC25A expression is increased in various tumor tissues and plays an important role in promoting tumor initiation and progression.^45^, ^46^, ^47^ Downregulation of CDC25A expression in GBM cells inhibited cell proliferation and DNA damage repair.^48^ CDC25A expression has been found to be upregulated by epidermal growth factor receptor (EGFR), which promotes cell growth in GBM.^49^

Although most splicing variants are probably nonfunctional,^50^ transcripts from nearly all human protein‐coding genes undergo one or more forms of alternative splicing.^51^ The most common type of alternative splicing is exon skipping, which is also known as cassette exons.^52^ CDC25A pre‐mRNA forms two transcripts via alternative splicing. CDC25A‐1 is the major transcript, while CDC25A‐2 is functionally defective because it lacks exon 6 through skipping.^39^ Our findings suggest that CDC25A exon 6 skipping correlates with the proliferative capacity of GBM monoclonal cells and the prognosis of GBM patients.

TDP‐43 is an RNA‐binding protein encoded by the TARDBP gene that regulates RNA splicing and stabilization.^53^ It has an nuclear localization signal (NLS (80–102 aa) and an nuclear export signal (NES) (239–250 aa) motif, which facilitate its nuclear localization.^54^ TDP‐43 primarily regulates biochemical reactions in the nucleus, but it can also shuttle between the nucleus and the cytoplasm.^55^ It binds to GU repeats of target RNAs through RRM1 and RRM2^56^ and regulates RNA splicing and stabilization of mRNAs encoding proteins involved in neuronal survival and neurodegenerative diseases, including Alzheimer's disease (AD) and amyotrophic lateral sclerosis (ALS).^57^, ^58^ TDP‐43 has been linked to the regulation of the expression of PAR3 and NUMB genes in triple‐negative breast cancer, which enhances proliferation and metastasis.^59^ In addition, TDP‐43, as a splicing repressor, leads to mis‐splicing of UNC13A in ALS.^38^ There is also evidence to suggest that TDP‐43 depletion causes exon 3 skipping of POLDIP3/SKAR in adult mouse brain samples.^60^In this study, we found that TDP‐43 binds ALKBH5 through its RRM1 structural domain, and the TDP‐43/ALKBH5 complex regulates CDC25A splicing through m^6^A modification thereby affecting the cell cycle G1/S phase transition. ALKBH5 may be present in DCD_4 and DCD_7 cells with different protein modifications that affect the amino acid sites at which ALKBH5 binds TDP‐43, leading to differences in the binding of ALKBH5 to TDP‐43 in DCD_4 and DCD_7 cells. We will explore this in our future studies.

The development of small molecule inhibitors targeting key proteins in the tumor signaling pathway shows significant therapeutic potential.^28^ Some of these inhibitors are currently in clinical use, while others are in various stages of clinical trials.^28^ Imatinib is employed for Philadelphia chromosome‐positive chronic granulocytic leukemia treatment, sorafenib has shown efficacy in advanced renal cell carcinoma treatment, and ongoing trials involve tivozanib targeting VEGFR for related diseases.^28^ Despite their promise, small molecule inhibitors face two primary clinical challenges: toxicity to normal tissues and the development of acquired resistance. Abnormalities in the MDM2‐p53 pathway, such as p53 mutations and MDM2 overexpression, are common among cancer cells, resulting in the loss of p53 tumor‐suppressive function.^61^ Consequently, multiple highly selective MDM2 inhibitors have been developed, with nine undergoing clinical trials.^62^ However, these inhibitors also exhibit toxicity to normal tissues, activating p53 not only in cancer cells but also in healthy cells like those in the spleen, bone marrow, and small intestine, potentially leading to adverse effects.^63^ Furthermore, prolonged MDM2 inhibitor treatment often results in acquired resistance.^64^ In this study, an inhibitor named TRAD01 was screened to target the interaction between TDP‐43 and ALKBH5. Its blood–brain barrier permeant was predicted using the SwissADME database, and its biological activity was confirmed in GBM cells and mouse models. Future research should focus on experimentally exploring the pharmacokinetics of TRAD01 to further substantiate its clinical translation potential.

## 4 CONCLUSION

In summary, we have identified a crucial epigenetic mechanism by which the TDP‐43/ALKBH5 complex contributes to GBM growth via its roles in m^6^A modification and alternative splicing. Specifically, TDP‐43 interaction with ALKBH5 facilitates ALKBH5 nuclear translocation and thus its binding to CDC25A pre‐mRNA in fast‐growing GBM cells.TDP‐43/ALKBH5 complex demethylates CDC25A pre‐mRNA, inhibiting its exon 6 skipping and preserving the expression of the oncogenic isoform CDC25A‐1, thereby promoting G1/S phase transition and growth of GBM cells. Finally, preclinical experiments support that targeting the binding of TDP‐43 and ALKBH5 might be a therapeutic strategy for GBM patients.

## 5 MATERIALS AND METHODS

### 5.1 Cell lines and primary cell cultures

The human glioma cell line U87MG was purchased from American Type Culture Collection (ATCC). The primary human GBM cell line NFHDCD was derived and cultured from a patient pathologically diagnosed with GBM (male, age 60, GBM) who underwent routine surgery at the Department of Neurosurgery of Nanfang Hospital (NFH, Guangzhou City, Guangdong Province, China). The cell lines were cultured as previously described.^65^

### 5.2 Animals

The nude mice used in this study were purchased from the Experimental Animal Center of Southern Medical University (4–6 weeks old, female, weighing approximately 15 g). The breeding conditions were maintained at a constant temperature (20–26°C) and humidity (50%–56%), in an environment that was free of specific pathogens and with clean air. The breeding equipment included mouse boxes and an air filtration hood, and corncob bedding, rodent feed, and drinking water were regularly replaced.

### 5.3 Sequence of siRNAs and shRNAs

The sequence of siRNAs and shRNAs of this study are available in Table S3.

### 5.4 Patients and tissue samples

Patients enrolled in this study were independently diagnosed with primary glioma by two pathologists in a double‐blinded manner according to the criteria of the 2021 WHO classification. They had undergone routine surgery at the Department of Neurosurgery of Nanfang Hospital (Guangzhou City, Guangdong Province, China) between 2013 and 2022 without radiotherapy or chemotherapy prior to surgery. The clinical characteristics of the samples used, such as age and gender, are shown in Tables S1 and S2.

### 5.5 Cell proliferation assay

Cell counting kit‐8 (CCK‐8), colony formation and EdU assays were performed as previously described.^65^, ^66^

### 5.6 Cell cycle analysis

Cells were fixed and then stained with PI/RNase Staining Buffer (BD Pharmingen) for 15 min at room temperature. Samples were acquired with a FACScan flow cytometer (BD Biosciences).

### 5.7 Xenograft assays

The animals were randomly grouped, and subcutaneous and intracranial xenograft experiments were performed as previously described.^67^

### 5.8 MeRIP‐seq

The m^6^A RNA‐Seq service was provided by Cloudseq Biotech Inc. Briefly, m^6^A RNA immunoprecipitation was performed with the GenSeq™ m^6^A‐MeRIP Kit (GenSeq Inc.) by following the manufacturer's instructions. Both the input samples without immunoprecipitation and the m^6^A IP samples were used for RNA‐seq library generation with the NEBNext® Ultra II Directional RNA Library Prep Kit (New England Biolabs, Inc.). The library quality was evaluated with a BioAnalyzer 2100 system (Agilent Technologies, Inc.). Library sequencing was performed on an Illumina NovaSeq 6000 instrument with 150 bp paired‐end reads.

### 5.9 MeRIP‐qPCR

The Foregene Total RNA Extraction Kit was used to isolate total RNA following the manufacturer's instructions. Briefly, mRNA was fragmented using RNA fragmentation reagent (Invitrogen, AM8740) for 15 min at 70°C. A small amount of fragmented RNA was left as input RNA and incubated for 2 h at 4°C in immunoprecipitation buffer (10 mM Tris–HCl, 150 mM NaCl, 0.1% Igepal CA‐630 and 400U RNasin Plus RNase inhibitor). The 7 mM m^6^A 5′‐monophosphate sodium salt was eluted for 1 h at 4°C with 5 mg glycogen, one‐tenth volume of 3 M sodium acetate, and 2.5 times volume of 100% ethanol (‐80°C). The enrichment of m^6^A was determined by qPCR analysis. The m^6^A primer sequences are shown in Table S4.

### 5.10 RNA immunoprecipitation (RIP)

RNA immunoprecipitation was performed using the Magna RIP™ RNA‐immunoprecipitation Kit (Millipore) according to the manufacturer's instructions. Briefly, magnetic beads coated with 5 µg TDP‐43 (Proteintech) were incubated overnight at 4°C with prefrozen cell lysates or nuclear extracts. The associated RNA–protein complexes were collected and washed 6 times, followed by proteinase K digestion and TRIzol extraction of RNA. Relative interactions between proteins and RNA were determined by qPCR and normalized to the input. The primer sequences are shown in Table S4.

### 5.11 Immunohistochemistry ()

IHC assays were performed on GBM samples or nude mouse xenograft tumor tissue to detect and score ALKBH5 and TDP‐43 expression. Paraffin‐embedded blocks were cut into 3 µm sections and deparaffinized and rehydrated. Antigen repair was performed by pressure cooking in citrate buffer (pH 6.0) for 5 min, followed by blocking endogenous peroxidase in 0.3% H_2_O_2_. After blocking with 5% bovine serum albumin (BSA) for 1 h, sections were incubated sequentially with primary antibody and horseradish peroxidase‐conjugated secondary antibody. Sections were covered with diaminobenzidine to visualize staining and then restained with hematoxylin prior to examination by microscopy.

### 5.12 HPLC–mass spectrometry analysis

Bands were cut out on sodium dodecyl‐sulfate polyacrylamide gel electrophoresis (‐) gels for proteolysis and peptide purification followed by mass spectrometry. Peptides were solubilized with sample lysis solution (0.1% formic acid). The mobile phase consisted of solvent A (0.1% formic acid, 2% acetonitrile/in water) and solvent B (0.1% formic acid, 80% acetonitrile). Peptides were separated with the following gradient: 0–5 min, 5%B; 5–45 min, 5%–50%B; 45–50 min, 50%–90%B; 50–55 min, 90%B, and all at a constant flow rate of 300 nL/min. The raw mass spectrometry files were converted by MM File Conversion software to obtain MGF format files, and then the database was retrieved using MASCOT (http://www.matrixscience.com/).

### 5.13 Extreme limiting dilution assay (ELDA)

Dissociated GSCs were seeded in 96‐well plates at a density of 5, 10, 20, 50, 100, or 200 cells per well, and each well was examined for the formation of tumorspheres after 7 days. Stem cell frequency was calculated using extreme limiting dilution analysis (http://bioinf.wehi.edu.au/software/elda/).

### 5.14 Protein–protein docking

Since there is no reported crystal structure of the TDP‐43 and ALKBH5 complex, for follow‐up research, we first docked TDP‐43 and ALKBH5 to predict the binding mode of TDP‐43 and ALKBH5. TDP‐43 (PDB ID: 4IUF) and ALKBH5 (PDB ID: 4O7X) were uploaded to the Zdock online server (http://zdock.umassmed.edu/) for protein‒protein docking, and we predicted the binding mode of TDP‐43 and ALKBH5 and finally selected the binding mode with the highest score for analysis.

### 5.15 Molecular dynamics simulation study

To elucidate the binding mechanism of the TDP‐43‐ALKBH5 complex, we retrieved the crystal structure data of the complex (PDB ID: 7U20) from the PDB database (https://www.rcsb.org/). The GROMACS software suite (version 2022.03) was employed to perform conventional molecular dynamics simulations to observe the dynamic conformational changes of the complex. The amber14sb force field was used for protein parameterization, and the TIP3P model was employed for water molecules. The complex was placed in an octahedral water box with an addition of 0.150 M NaCl to neutralize the charge. The system was first energy‐minimized for 50,000 steps using the steepest descent method, followed by equilibration simulations in the canonical ensemble (NVT) and constant‐pressure, constant‐temperature (NPT) ensembles for 50,000 steps each, maintaining a temperature of 300 K and pressure of 1 bar. After equilibration, the system reached a steady state at the set temperature and pressure, followed by a 100‐ns unrestrained simulation, during which energy and coordinate data were collected every 20 ps.

### 5.16 Free energy calculation and residue decomposition

The molecular mechanics/generalized Born surface area (MM‐GBSA) method, widely applied for estimating binding free energies in drug research due to its reliability, was utilized in this study. Using the gmx_MMPBSA tool embedded within GROMACS, we performed MM‐PB (GB) SA free energy calculations. To intricately analyze the molecular mechanism of binding between ALKBH5 and TDP‐43, the contributions of individual residues to the binding free energy were meticulously evaluated with the gmx_MMPBSA tool.

### 5.17 Virtual screening

Molecular docking and virtual screening were executed using the Schrodinger software suite, including the Glide module. The TDP‐43‐ALKBH5 complex was the target for the virtual screening against a small molecule library. Docking grids were constructed based on the potential active sites of TDP‐43. The screening process followed the stepwise protocol in the Glide module. The database from ChemDiv (https://www.chemdiv.com/) served as the input for this screening. Resultant docking poses were analyzed based on their scores, and the top‐ranked compounds were further scrutinized.

### 5.18 Statistical analysis

Experiments were conducted using a minimum of three replicates per experimental group. Representative data from at least two replicate experiments are described. Statistical analysis was performed using GraphPad Prism 8. Statistical graph was plotted by GraphPad Prism 8 and https://www.bioinformatics.com.cn. Student's *t*‐tests, Mann–Whitney *U*‐tests, one‐way analysis of variance (ANOVA), Pearson correlation analyses, Kaplan–Meier analyses, log‐rank tests, Cox's proportional hazards regression model, and *χ* ^2^‐tests were used to analyze the corresponding data. Asterisks denote statistical significance (ns, no significance; *p < 0.05; **p < 0.01; ***p < 0.001; ****p < 0.0001).

## AUTHOR CONTRIBUTIONS

Yunxiao Zhang and Sidi Xie conceived the project, designed and performed the research, analyzed and interpreted the data, and wrote the manuscript. Weizhao Li and Junwei Gu conducted supplementary experiments. Xi‐an Zhang performed the research and analyzed and interpreted the data. Bowen Ni, Ziyu Wang, Runwei Yang, Haimin Song, Yaxuan Zhong, and Peiting Huang provided assistance in some experiments. Jinyao Zhou, Yongfu Cao, and Jing Guo provided material support. Yawei Liu, Songtao Qi, and Hai Wang conceived and designed the study, wrote the manuscript, and provided study supervision. All the authors have read and approved the final manuscript.

## CONFLICT OF INTEREST STATEMENT

The authors declare no conflicts of interest.

## ETHICS STATEMENT

The experimental protocol was established according to the ethical guidelines of the Helsinki Declaration and was approved by the Ethics Committee of Southern Medical University, China (No. NFEC‐BPE‐207). Written informed consent was obtained from individual participants or their guardians. All animal experiments were performed following approval of the Institutional Animal Care and Use Committee of Southern Medical University, China (No. IACUC‐LAC‐20240311‐003).

## Supporting information



Supporting Information

## Data Availability

All data supporting the findings of this study are available from the authors upon reasonable request.
