# Supplementary material for: TDP‐43/ALKBH5‐mediated m6A modification of CDC25A mRNA promotes glioblastoma growth by facilitating G1/S cell cycle transition
Source: MedComm (2020). 2025 Feb 18;6(3):e70108. doi: 10.1002/mco2.70108 (PMC11836349; doi:10.1002/mco2.70108)
Supplement: Supplementary file 1 — Supporting Information [file MCO2-6-e70108-s001.docx]

**TDP-43/ALKBH5-mediated m^6^A modification of CDC25A mRNA promotes glioblastoma growth by facilitating G1/S cell cycle transition**

Yunxiao Zhang^1,2,#^, Sidi Xie^1,2,#^, Weizhao Li^1,2,#^, Junwei Gu^3,#^, Xi-an Zhang^1,2^, Bowen Ni^4^, Ziyu Wang^4^, Runwei Yang^1,2^, Haimin Song^5^, Yaxuan Zhong^6^, Peiting Huang^7^, Jinyao Zhou^8^, Yongfu Cao^9^, Jing Guo^10^, Yawei Liu^4,*^, Songtao Qi^1,2,*^, Hai Wang^1,2,*^

1 Department of Neurosurgery, Nanfang Hospital, Southern Medical University, Guangzhou, China

2 Department of Neurosurgery, Institute of Brain Diseases, Nanfang Hospital of Southern Medical University, Guangzhou, China

3 The First People's Hospital of Xiushui County, Jiujiang, China

4 Department of Neurosurgery & Medical Research Center, Shunde Hospital, Southern Medical University, Shunde, China

5 Department of Neurosurgery, The First Affiliated Hospital of Gannan Medical University, Ganzhou, China

6 School of the First Clinical Medicine, Gannan Medical University, Ganzhou, China

7 Department of Neurology, Guangdong Provincial People's Hospital, Southern Medical University, Guangzhou, Guangdong, China

8 Department of Neurosurgery, Dongguan Tungwah Hospital, Dongguan, China

9 Neurosurgery, Key Laboratory of Biological Targeting Diagnosis, Therapy and Rehabilitation of Guangdong Higher Education Institutes, The Fifth Affiliated Hospital of Guangzhou Medical University, Guangzhou, China

10 Epilepsy Center, Guangdong Sanjiu Brain Hospital, Guangzhou, China

# Yunxiao Zhang, Sidi Xie, Weizhao Li, and Junwei Gu have contributed equally to this work and share first authorship.

*Correspondence

Yawei Liu, Department of Neurosurgery & Medical Research Center, Shunde Hospital, Southern Medical University, Shunde, 528300, China.

Email: [liuyawei@smu.edu.cn](mailto://liuyawei@smu.edu.cn)

Songtao Qi and Hai Wang, Department of Neurosurgery, Nanfang Hospital, Southern Medical University, Guangzhou, 510515, China.

Email: [qisongtaonfyy@126.com](mailto://qisongtaonfyy@126.com) and [shandongwanghai@163.com](mailto://shandongwanghai@163.com)

**
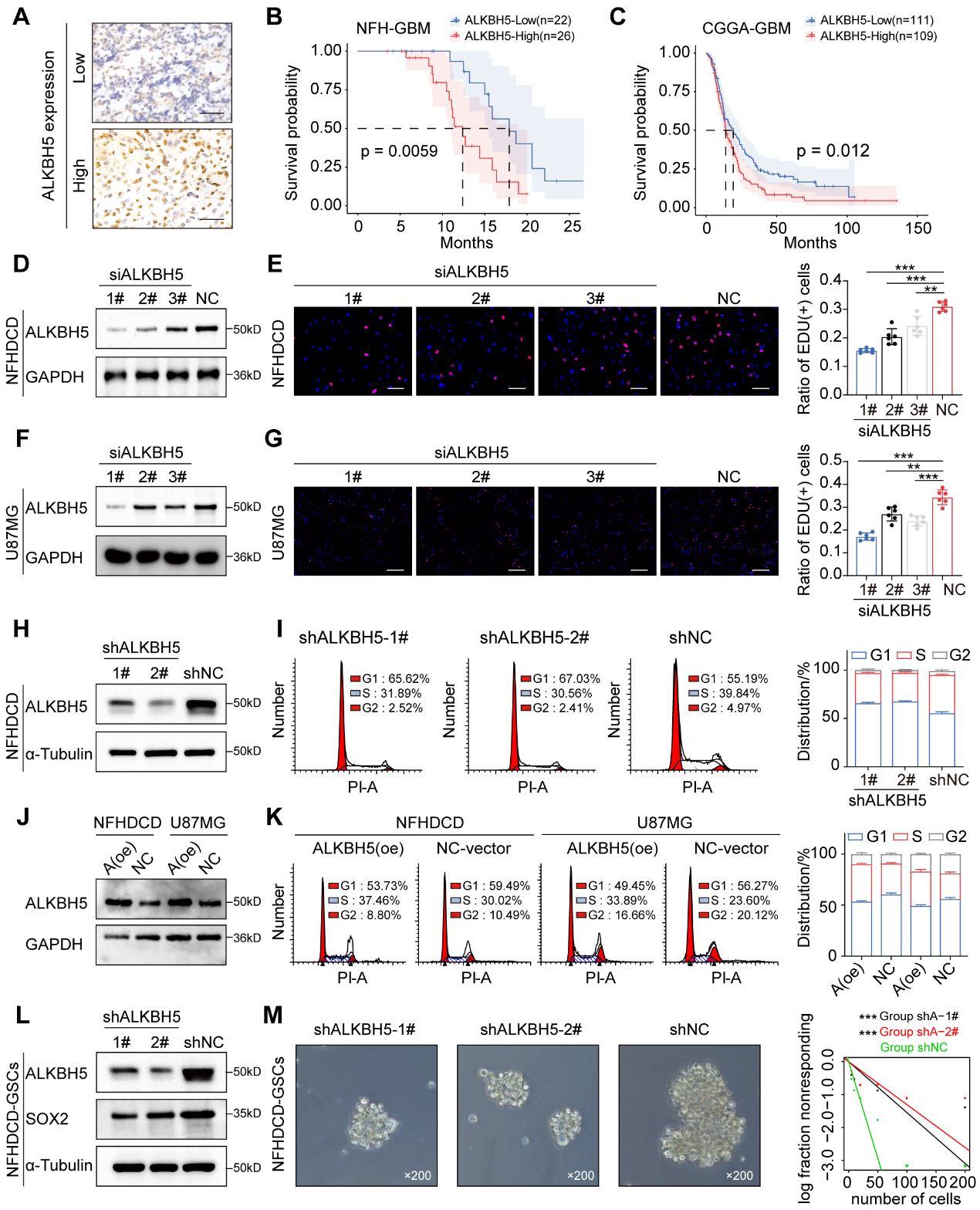
**

**Figure S1. A** IHC staining images for ALKBH5 in representative GBM samples obtained from NFH. Scale bars = 50 μm. **B** Kaplan–Meier survival curve of NFH-GBM patients stratified by the overall protein expression of ALKBH5 (according to IHC data). **C** Kaplan–Meier survival analysis of CGGA GBM data stratified by the total mRNA expression of ALKBH5. **D** WB analysis of ALKBH5 and GAPDH in NFHDCD cells after transfection with the indicated siRNA. **E** EdU assay showing different cell proliferation rates in siALKBH5- and NC-treated NFHDCD cells. Scale bars=100 μm. **F** WB analysis of ALKBH5 and GAPDH in U87MG cells after transfection with the indicated siRNA. **G** EdU assay showing different cell proliferation rates in siALKBH5- and NC-treated U87MG cells. Scale bars=200 μm. **H** WB analysis of ALKBH5 and α-Tubulin in NFHDCD cells after transfection with the indicated shRNA. **I** Cell cycle flow analysis in the NFHDCD cells after the indicated shRNA transfection. **J** WB analysis of ALKBH5 and GAPDH in NFHDCD and U87 cells treated with ALKBH5 (oe) and NC vector. **K** Cell cycle flow analysis in the NFHDCD and U87 cells treated with ALKBH5 (oe) and NC vector. **L** WB analysis of ALKBH5 and α-Tubulin in NFHDCD-GSCs cells after transfection with the indicated shRNA. **M** Left: Morphology of NFHDCD-GSCs cells after ALKBH5 depletion. Right: ELDA assay analysis in NFHDCD-GSCs cells after the indicated shRNA transfection. Data are expressed as the mean ± SD. **, P < 0.01; ***, P < 0.001.

**
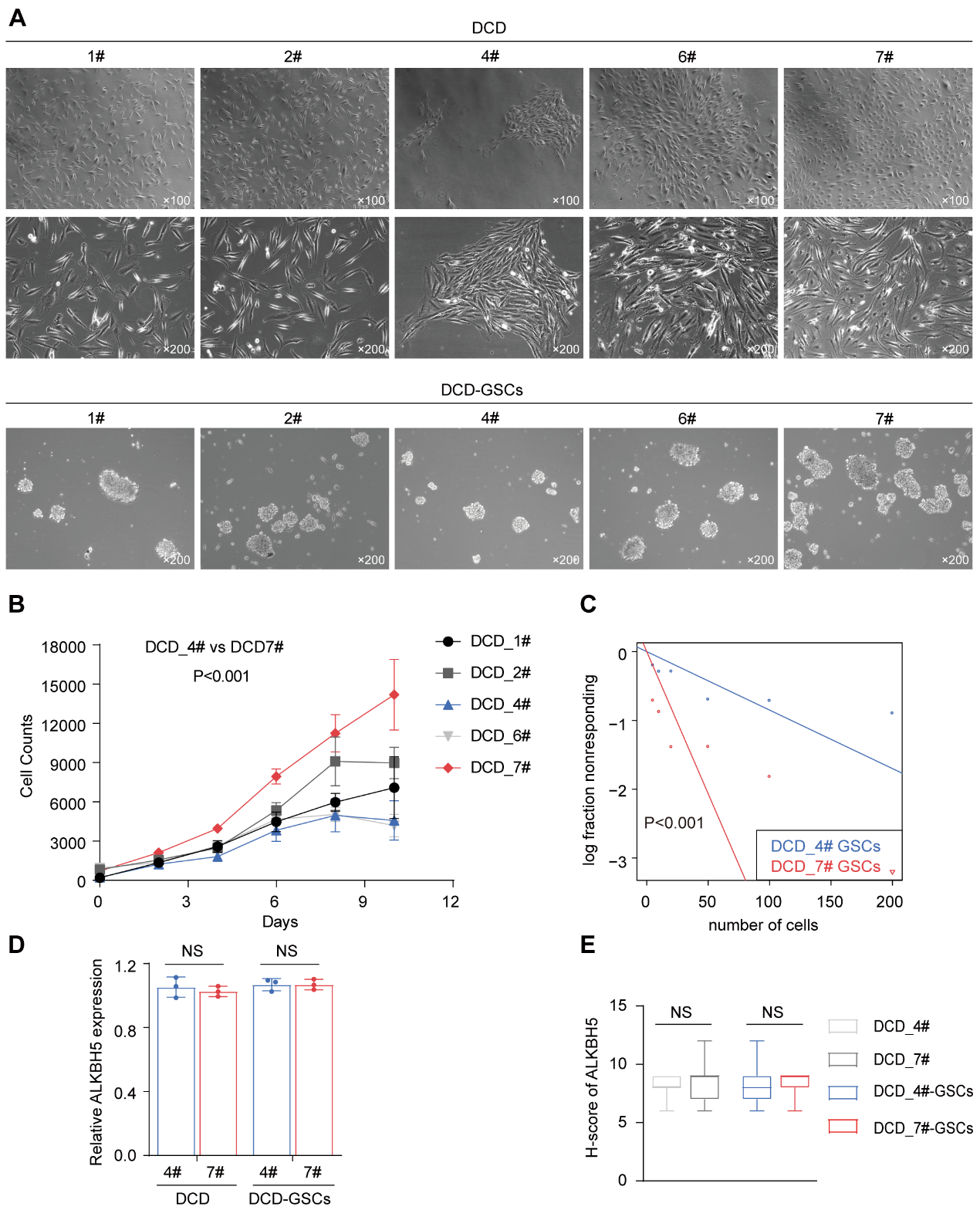
**

**Figure S2. A** Morphology of primary monoclonal cells and their GSCs. **B** CCK8 assay showing different cell proliferation rates in primary monoclonal cells. **C** ELDA analysis in DCD_4# and DCD_7# GSCs. **D** Statistical graphs for WB analysis of ALKBH5 and GAPDH. **E** Statistical graphs for ALKBH5 IHC staining scores. Data are expressed as the mean ± SD.


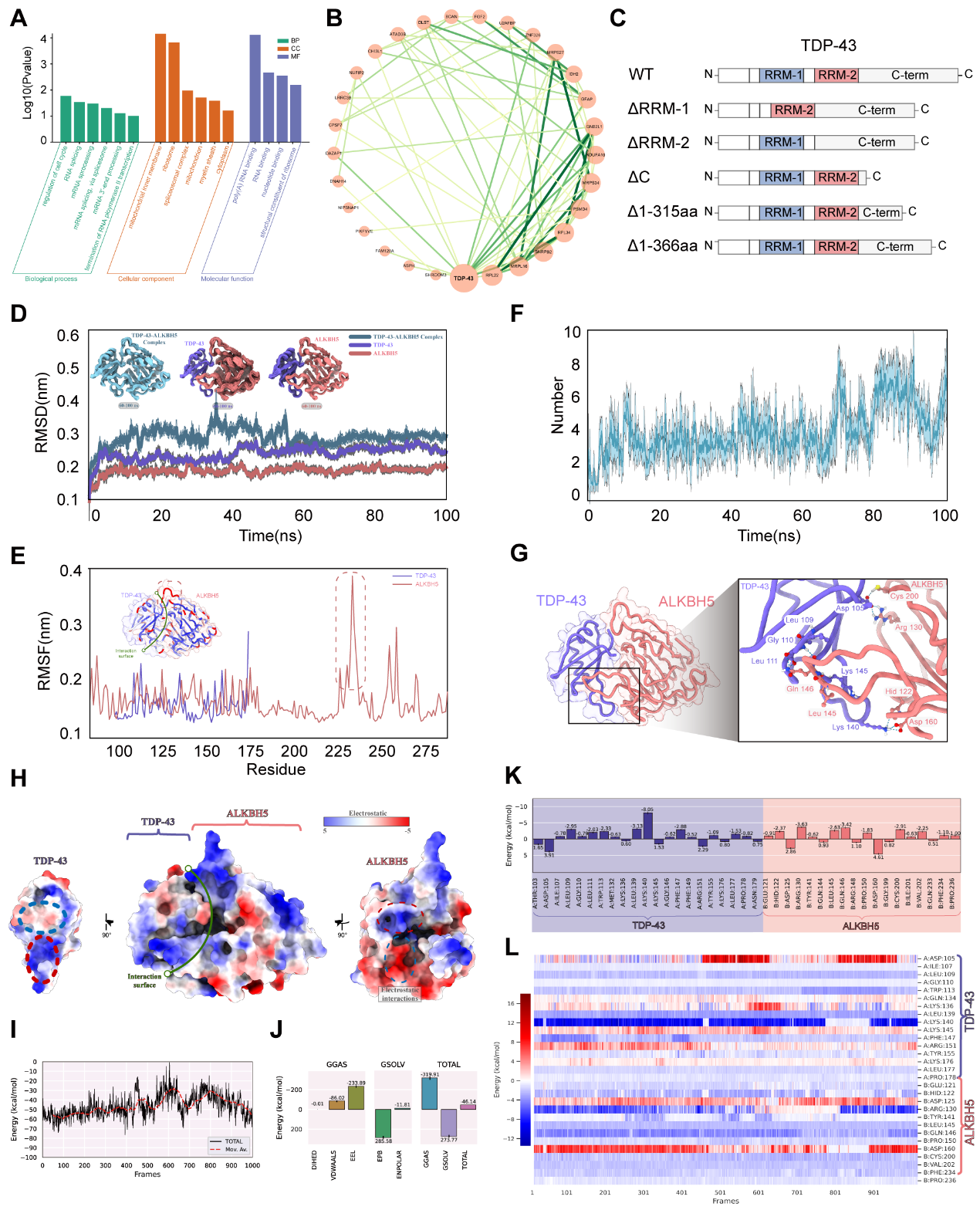


**Figure S3. A** GO analysis of the interacting proteins of ALKBH5 in DCD_7# cells. **B** The PPI network analysis of the interacting proteins of ALKBH5 in DCD_7# cells. **C** Schematic representation of the constructed TDP-43 truncates. **D** RMSD of 100ns molecular dynamics simulation of TDP-43 and ALKBH5 complex. Curves: dark green: RMSD of TDP-43 and ALKBH5 complex. pink and purple: RMSD of TDP-43 and ALKBH5 individual structures. Structures: dark green, pink and purple: TDP-43 and ALKBH5 complex taken at 1ns intervals in 60-100ns molecular dynamics simulations and individually superimposed centered on TDP-43 and ALKBH5 complex, TDP-43 and ALKBH5, respectively. **E** RMSF and B-factor of TDP-43 and ALKBH5 complexes. Curves: blue and pink color represent the RMSF of TDP-43 and ALKBH5, respectively. Structures: blue and pink colors represent the B factors of TDP-43 and ALKBH5, respectively. **F** Number of hydrogen bonds formed by TDP-43 and ALKBH5 complex during 100ns molecular dynamics simulations. **G** Schematic representation of the interaction between TDP-43 and ALKBH5 complex at 100ns of molecular dynamics simulation. **H** Potential surfaces of TDP-43 and ALKBH5 complex. **I** The last 10ns of the trajectory of the molecular dynamics of the TDP-43 and ALKBH5 complexes were extracted (1000 frames in total) and the free energy of binding between TDP-43 and ALKBH5 was calculated for each frame. **J** The free energy of binding between TDP-43 and ALKBH5 decomposes into the contribution terms of GGAS and GSOLV. **K** The free energy of binding between TDP-43 and ALKBH5 is decomposed into a contribution term for amino acids on the interaction surface. **L** The free energy of binding between TDP-43 and ALKBH5 is decomposed into the contribution terms of the amino acids on the interaction surface for each frame.

**
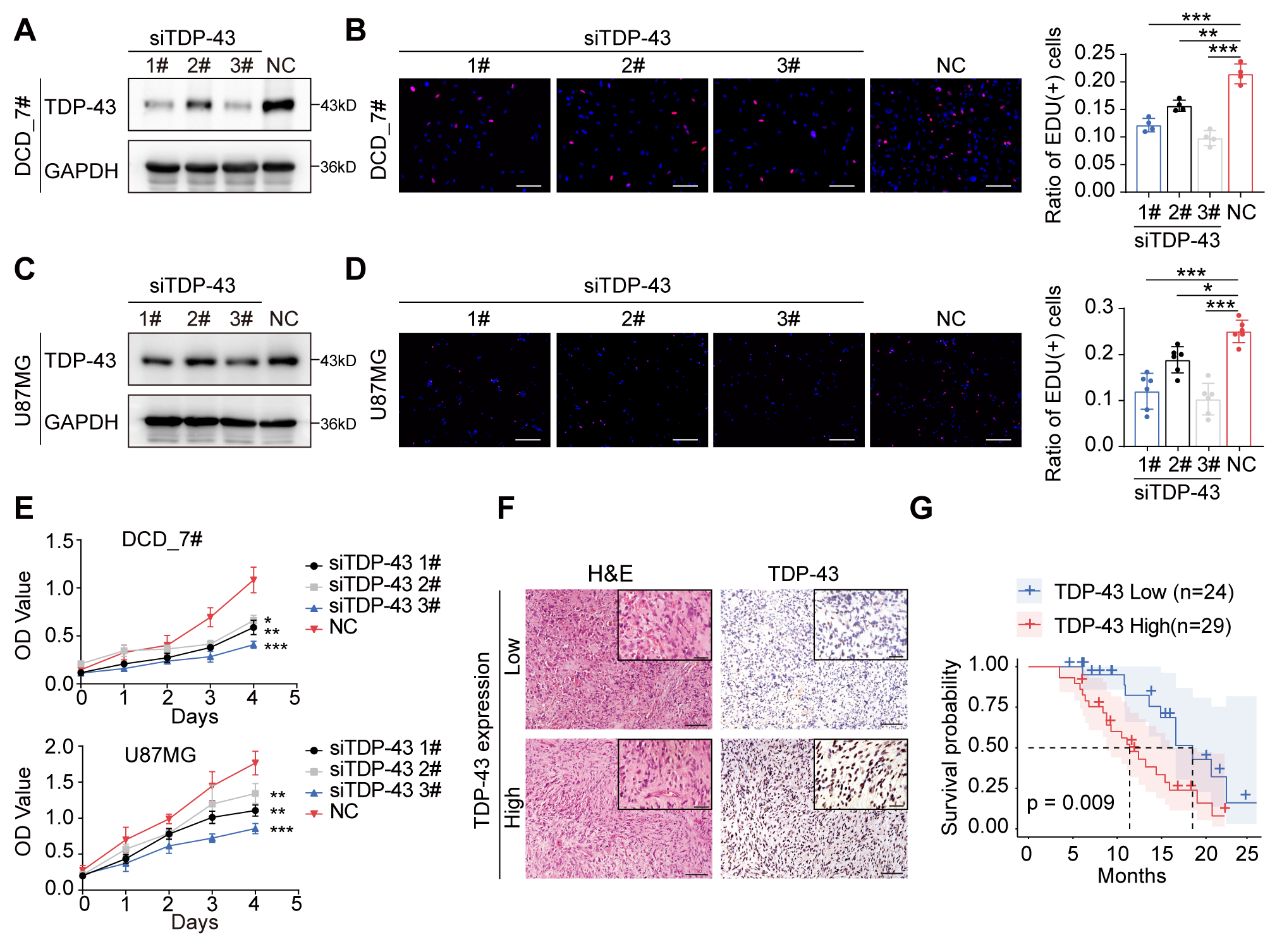
**

**Figure S4. A** WB analysis of TDP-43 and GAPDH in DCD_7# cell lines after transfection with the indicated siRNAs. **B** EdU assays in DCD_7# cells after transfection with the indicated siRNAs. Scale bars=100 μm. **C** WB analysis of TDP-43 and GAPDH in U87MG cell lines after transfection with the indicated siRNAs. **D** EdU assays in U87MG cells after transfection with the indicated siRNAs. Scale bars=200 μm. **E** CCK8 assay in DCD_7# and U87MG cells after transfection with the indicated siRNAs. **F** H&E and IHC staining images of representative GBM samples obtained from NFH. Scale bars = 50 μm and 100 μm. **G** Kaplan–Meier survival curve of NFH-GBM patients stratified by TDP-43 expression (according to IHC data). Data are expressed as the mean ± SD. *, P < 0.05; **, P < 0.01; ***, P < 0.001.


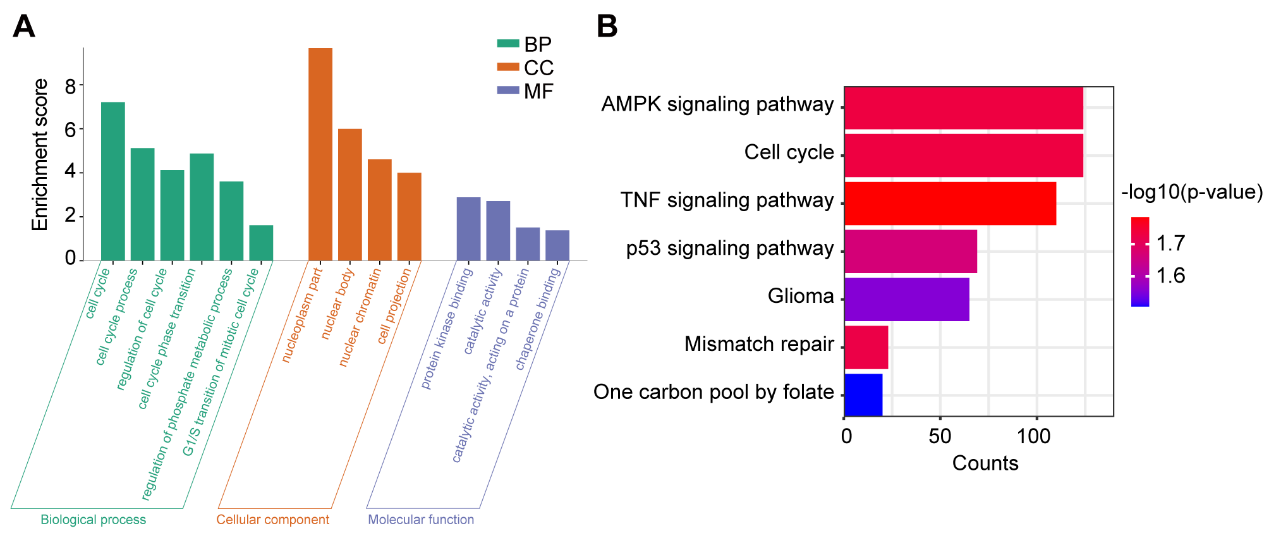


**Figure S5. A** GO analysis of m6A level upstream genes in DCD_4# compared to DCD_7# cells. **B** KEGG analysis of m^6^A level upstream genes in DCD_4# compared to DCD_7# cells.


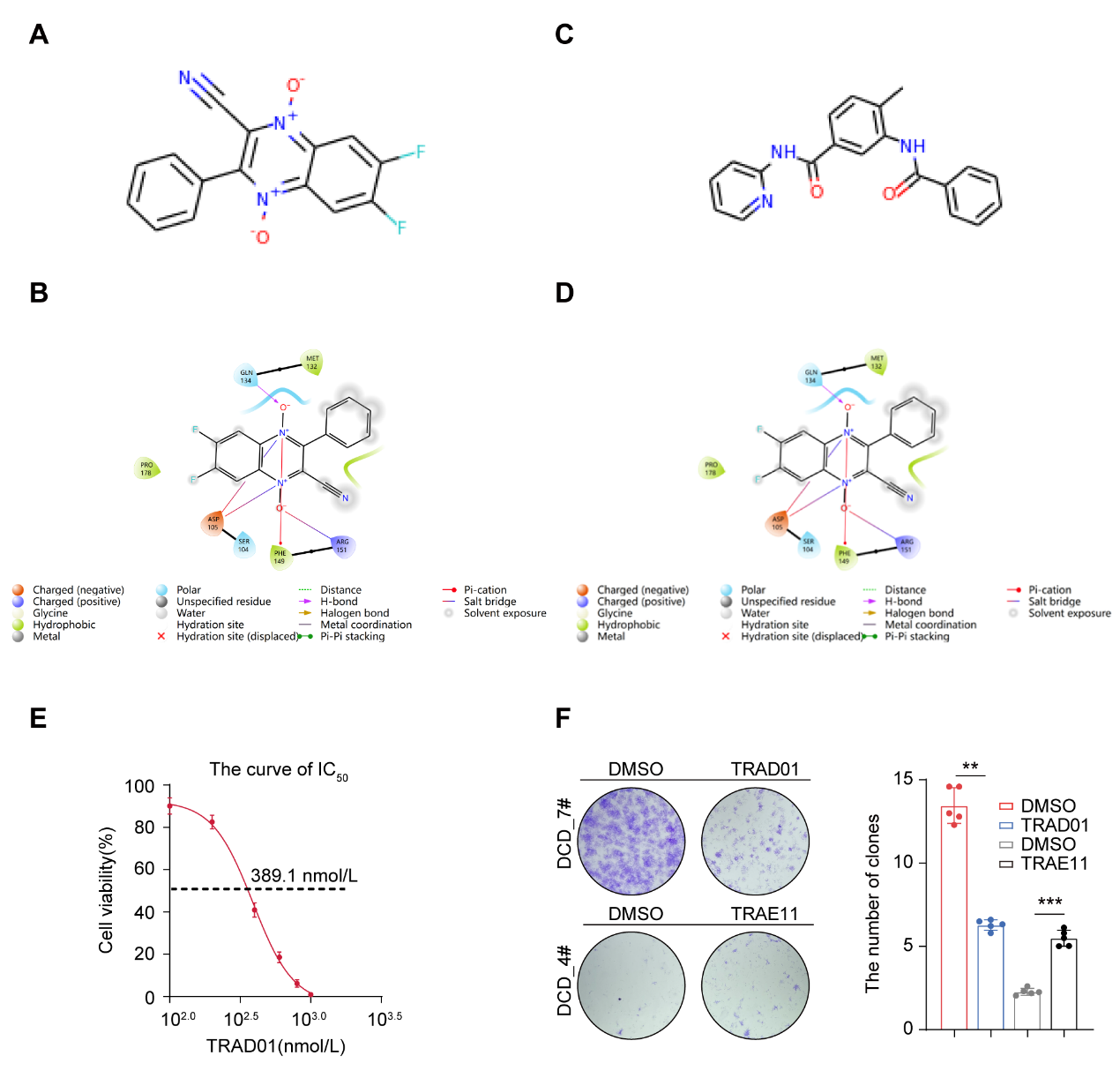


**Figure S6. A** Structure of TRAD01. **B** 2D representation of TRAD01 binding pocket. **C** Structure of TRAE11. **D** 2D representation of TRAE11 binding pocket. **E** IC_50_ curves of DCD_7# cells after 24 hours of TRAD01 treatment. **F** Plate cloning assay in DCD_7# cells after TRAD01 treatment and DCD_4# cells after TRAE11 treatment. Data are expressed as the mean ± SD. **, P < 0.01; ***, P < 0.001.

**Table S1.** Clinical Characteristics of the 48 glioblastoma specimens in IHC assay of ALKBH5

| Characteristics | ALKBH5 expression  (According to IHC staining score) | | Total  (n=48) | P Value |
| --- | --- | --- | --- | --- |
|  | Low (n=22) | High (n=26) |  |  |
| **Gender** |  |  |  |  |
| Female | 10(45.5%) | 9(34.6%) | 19(39.6%) | 0.4442 |
| Male | 12(54.5%) | 17(65.4%) | 29(60.4%) |  |
| **Age** |  |  |  |  |
| <=50 | 11(50.0%) | 14(53.8%) | 25(52.1%) | 0.7904 |
| >50 | 11(50.0%) | 12(46.2%) | 23(47.9%) |  |
| **IDH1 Mutation Status** |  |  |  |  |
| Mutant | 8(36.4%) | 10(38.5%) | 18(37.5%) | 0.8811 |
| Wild-type | 14(63.6%) | 16(61.5%) | 30(62.5%) |  |
| **MGMT Expression**  MGMT+ | 13(59.1%) | 15(57.7%) | 28(58.3%) | 0.9220 |
| MGMT- | 9(40.9%) | 11(42.3%) | 20(41.7%) |  |

**Table S2.** Clinical Characteristics of the 53 glioblastoma specimens in IHC assay of TDP-43

| Characteristics | TDP-43 expression  (According to IHC staining score) | | Total  (n=53) | P Value |
| --- | --- | --- | --- | --- |
|  | Low (n=24) | High (n=29) |  |  |
| **Gender** |  |  |  |  |
| Female | 13(54.2%) | 13(44.8%) | 26(49.1%) | 0.4984 |
| Male | 11(48.8%) | 16(55.2%) | 27(50.9%) |  |
| **Age** |  |  |  |  |
| <=50 | 9(37.5%) | 11(37.9%) | 20(37.7%) | 0.9743 |
| >50 | 15(62.5%) | 18(62.1%) | 33(62.3%) |  |
| **IDH1 Mutation Status** |  |  |  |  |
| Mutant | 10(41.7%) | 15(51.7%) | 25(47.2%) | 0.4653 |
| Wild-type | 14(58.3%) | 14(48.3%) | 28(52.8%) |  |
| **MGMT Expression**  MGMT+ | 12(50.0%) | 10(34.5%) | 22(41.5%) | 0.2538 |
| MGMT- | 12(50.0%) | 19(65.5%) | 31(58.5%) |  |

**TableS3.** Sequence of siRNAs and shRNAs

| **siRNAs or shRNAs** | **Sequence (5'-3')** |
| --- | --- |
| ALKBH5 siRNA1# | ACAAGUACUUCUUCGGCGA |
| ALKBH5 siRNA2# | GCGCCGUCAUCAACGACUA |
| ALKBH5 siRNA3# | CUGAGAACUACUGGCGCAA |
| Negative control siRNA | UUCUCCGAACGUGUCACGUTT |
| TDP-43 siRNA1# | GCUCUAAUUCUGGUGCAGCAAdTdT |
| TDP-43 siRNA2# | GCUUUGGCUCAAGCAUGGAUUdTdT |
| TDP-43 siRNA3# | GCAAUAGACAGUUAGAAAGAAdTdT |
| Negative control siRNA | UUCUCCGAACGUGUCACGUTT |
| LV-ALKBH5-1# shRNA | GCGCCGUCAUCAACGACUA |
| LV-ALKBH5-2# shRNA | ACAAGUACUUCUUCGGCGA |
| LV-Negative control | GAAUACGUACCCCAUUAUA |
| LV-TDP-43-1# shRNA | GCUCUAAUUCUGGUGCAGCAA |
| LV-TDP-43-2# shRNA | GCTTTGGCTCAAGCATGGATT |
| LV-Negative control | CCTAAGGTTAAGTCGCCCTCG |

**TableS4.** Primer sequences

| **Gene** | **Primer sequence (5'-3')** |
| --- | --- |

| CDC25A | Forward primer | GAAATGCTGCTGGCCAAATAG |
| --- | --- | --- |
|  | Forward primer | CGAGGTAGTCCCTGGGATAATA |
| RAD51 | Forward primer | TGGCAGTGGCTGAGAGGTATG |
|  | Reverse primer | GGTCTGGTGGTCTGTGTTGAAC |
| PRKAA2 | Forward primer | GGCACCCTCCCATTTGATGATG |
|  | Reverse primer | GAGAGTGGCGACAGAACGATTG |
| TP53INP1 | Forward primer | AGAAGAGGAGGACATCAGTGAAGAG |
|  | Reverse primer | TTGTATCAGCCAAGCACTCAAGAG |
| GAPDH | Forward primer | GTCAACGGATTTGGTCGTATTG |
|  | Reverse primer | TGGAAGATGGTGATGGGATTT |
| CDC25A m^6^A peak | Forward primer | CGCCACGTTTGTTTGGATTTA |
|  | Forward primer | GTCAAACACAAACACGACTCC |
| RAD51 m^6^A peak | Forward primer | GCCAGAGACCGAGCCCTAAGG |
|  | Reverse primer | GCTCCACTTCTCTACTCGCTTGC |
| PRKAA2 m^6^A peak | Forward primer | ACCAGCTTGCAGTGGCTTATCATC |
|  | Reverse primer | GGAGGACTAGAGGCGAGGTAGAAC |
| TP53INP1 m^6^A peak | Forward primer | CGGGCAGGGCTGATGAACAAC |
|  | Reverse primer | AGCCTTCGTCGCCAAAATCTTACC |

**Supplemental Materials**

| **Reagents or chemicals** | **Source** | **Identifier** |
| --- | --- | --- |
| anti-ALKBH5（WB,IP） | Proteintech | Cat#16837-1-AP |
| anti-ALKBH5（IF,RIP） | Millipore | Cat#ABE547 |
| anti-GAPDH | Proteintech | Cat#10494-1-AP |
| anti-TDP-43 (WB, IP) | Proteintech | Cat#18280-1-AP |
| anti-TDP-43 (IF) | Proteintech | Cat#60019-2-Ig |
| anti-Flag | Sigma-Aldrich | Cat#F3165 |
| Normal rabbit IgG antibody | Proteintech | Cat#B900610 |
| anti-m6A | Millipore | Cat#MABE1006 |
| anti-CDK2 | Cell Signaling Technology | Cat#2546 |
| anti-p-CDK2  TRAD01(2-Quinoxalinecarbonitrile, 6,7-difluoro-3-phenyl-, 1,4-dioxide) | Abcam  HwaGen | Cat#ab68265  CAS# 504423-47-6 |
| HRP AffiniPure Goat Anti-Mouse IgG（H+L） | Fdbio science | FDM007 |
| HRP AffiniPure Goat Anti-Rabbit IgG（H+L） | Fdbio science | FDR007 |
| PI/RNase Staining Buffer | BD Pharmingen | Cat#550825 |
| Cell Light EdU Apollo 564 In Vitro Kit | RIBOBIO | Cat#C10310-1 |
| Cell counting kit-8 | Bimake | Cat#B34304 |
| Magna RIP™ RNA-Binding Protein Immunoprecipitation Kit | Millipore | Cat#17-700 |
| Immunoprecipitation buffer | Sigma-Aldrich | Cat#I8896 |
| RNasin Plus RNase inhibitor | Promega | Cat#N2611 |
| N6-methyladenosine 5'-monophosphate sodium salt | Sigma-Aldrich | Cat#M2780 |
| Cell Lysis Buffer for IP | Abclonal | Cat#RM00020 |
| Lipofectamine 2000 | Invitrogen | Cat#52887 |
| Protein A/G Magetic Beads for IP | Bimake | Cat#23201 |
| TRAD01/TRAE11 | Hwagen |  |

**Supplemental Methods**

**Immunohistochemistry (IHC)**

IHC assays were performed on GBM samples or nude mouse xenograft tumor tissue to detect and score ALKBH5 and TDP-43 expression. Paraffin-embedded blocks were cut into 3 μm sections and de-paraffinized and re-hydrated. Antigen repair was performed by pressure cooking in citrate buffer (pH 6.0) for 5 min, followed by blocking endogenous peroxidase in 0.3% H_2_O_2_. After blocking with 5% bovine serum albumin (BSA) for 1 hour, sections were incubated sequentially with primary antibody and horseradish peroxidase-conjugated secondary antibody. Sections were covered with diaminobenzidine to visualize staining and then restained with hematoxylin prior to examination by microscopy.

**HPLC-Mass spectrometry analysis**

The raw mass spectrometry files were converted by MM File Conversion software to obtain MGF format files, and then the database was retrieved using MASCOT (http://www.matrixscience.com/).

**Limiting dilution assay**

Dissociated GSCs were seeded in 96-well plates at a density of 5, 10, 20, 50, 100 or 200 cells per well, and each well was examined for the formation of tumorspheres after 7 days. Stem cell frequency was calculated using extreme limiting dilution analysis (<http://bioinf.wehi.edu.au/software/elda/>).

**Protein‒protein docking**

Since there is no reported crystal structure of the TDP-43 and ALKBH5 complex, for follow-up research, we first docked TDP-43 and ALKBH5 to predict the binding mode of TDP-43 and ALKBH5. TDP-43 (PDB ID: 4IUF) and ALKBH5 (PDB ID: 4O7X) were uploaded to the Zdock online server (http://zdock.umassmed.edu/) for protein‒protein docking, and we predicted the binding mode of TDP-43 and ALKBH5 and finally selected the binding mode with the highest score for analysis.

**Molecular Dynamics Simulation Study**

To elucidate the binding mechanism of the TDP-43-ALKBH5 complex, we retrieved the crystal structure data of the complex (PDB ID: 7U20) from the PDB database (https://www.rcsb.org/). The GROMACS software suite (version 2022.03) was employed to perform conventional molecular dynamics simulations to observe the dynamic conformational changes of the complex. The amber14sb force field was used for protein parameterization, and the TIP3P model was employed for water molecules. The complex was placed in an octahedral water box with an addition of 0.150 M NaCl to neutralize the charge. The system was first energy-minimized for 50,000 steps using the steepest descent method, followed by equilibration simulations in the NVT and NPT ensembles for 50,000 steps each, maintaining a temperature of 300 K and pressure of 1 bar. After equilibration, the system reached a steady state at the set temperature and pressure, followed by a 100-ns unrestrained simulation, during which energy and coordinate data were collected every 20 ps.

**Free Energy Calculation and Residue Decomposition**

The MM-GBSA method, widely applied for estimating binding free energies in drug research due to its reliability, was utilized in this study. Using the gmx_MMPBSA tool embedded within GROMACS, we performed MM-PB (GB) SA free energy calculations. To intricately analyze the molecular mechanism of binding between ALKBH5 and TDP-43, the contributions of individual residues to the binding free energy were meticulously evaluated with the gmx_MMPBSA tool.

**Virtual Screening**

Molecular docking and virtual screening were executed using the Schrodinger software suite, including the Glide module. The TDP-43-ALKBH5 complex was the target for the virtual screening against a small molecule library. Docking grids were constructed based on the potential active sites of TDP-43. The screening process followed the stepwise protocol in the Glide module. The database from ChemDiv (https://www.chemdiv.com/) served as the input for this screening. Resultant docking poses were analyzed based on their scores, and the top-ranked compounds were further scrutinized.
